# Apoptosis and cuproptosis Co-activated Copper-based metal-organic frameworks for cancer therapy

**DOI:** 10.1186/s12951-024-02828-3

**Published:** 2024-09-06

**Authors:** Kun Li, Leilei Wu, Han Wang, Zi Fu, Jiani Gao, Xiucheng Liu, Yongfei Fan, Xichun Qin, Dalong Ni, Jing Wang, Dong Xie

**Affiliations:** 1 Department of Thoracic Surgery, Shanghai Pulmonary Hospital, School of Medicine, Tongji University, Shanghai, 200433 P. R. China; 2 Department of Thoracic Surgery, Hangzhou Institute of Medicine (HIM), Zhejiang Cancer Hospital, Chinese Academy of Sciences, Hangzhou, 310005 P. R. China; 3 Department of Orthopaedics, Shanghai Key Laboratory for Prevention and Treatment of Bone and Joint Diseases, Shanghai Institute of Traumatology and Orthopaedics, Ruijin Hospital, Shanghai Jiao Tong University School of Medicine, Shanghai, 200025 P. R. China; 4 Suzhou Institute of Biomedical Engineering and Technology, Chinese Academy of Science, Suzhou, 215163 P. R. China; 5 Department of Radiology, Shanghai Fourth People’s Hospital, School of Medicine, Tongji University, Shanghai, 200434 P. R. China

**Keywords:** Cuproptosis, Apoptosis, Cytoskeletal structure, Metal-organic frameworks, Nanomedicine

## Abstract

Lung cancer, predominantly non-small cell lung cancer (NSCLC), remains a significant global health challenge, with limited therapeutic options for patients with *KRAS*-mutated tumors. Herein, a copper-based metal-organic framework (Cu-MOF) was applied as a novel cuproptosis-mediated nanoplatform for lung cancer therapy. Cu-MOF would disassemble and liberate copper ions under the acidic microenvironment of lysosomes of cancer cells, initiating a cascade of cellular events. The released copper ions catalyzes the Fenton reaction, generating hydroxyl radicals that induce oxidative damage, leading to cytoskeletal disruption and activation of caspase-3, ultimately triggering apoptosis. Simultaneously, with the mediation of the key regulatory factor FDX1, we found that the copper ions binding to the mitochondrial protein DLAT could result in the loss of iron-sulfur cluster proteins and aggregation of lipoylated proteins, which culminated in proteotoxic stress-induced cuproptosis. The pronounced anti-tumor effects of Cu-MOF with apoptosis and cuproptosis were confirmed both in vitro and in vivo experiments. Such dual induction of apoptosis and cuproptosis by Cu-MOF presents a promising therapeutic strategy for NSCLC, particularly for *KRAS*-mutated tumors, and expands potential applications of Cu-based nanomateirals for other cancers.

**Supplementary Information:**

The online version contains supplementary material available at 10.1186/s12951-024-02828-3.

## Introduction

Lung cancer is the second most common cancer in the world and has the highest mortality rate, with non-small cell lung cancer (NSCLC) accounting for more than 80% of cases [1]. Kirsten rat sarcoma viral oncogene homologue (*KRAS*) are the most commonly mutated oncogenes in NSCLC, accounting for approximately 30% [2]. The *KRAS* G12C (*KRAS*^*G12C*^) variant is the most common isoform (about 40% of *KRAS* mutant), followed by G12V, G12D, G12A, codon 13 and Q61 mutations [3]. However, recent studies have shown that *KRAS*-mutated NSCLCs are highly heterogeneous and that most of these patients are generally resistant to immunotherapies [4, 5]. Although the new *KRAS*^*G12C*^-targeted small molecule (G12Ci) has the ability to inhibit *KRAS* signaling and generate objective responses in this patient population, only a minority of *KRAS*^*G12C*^ NSCLC patients have responded to these treatments. Moreover, even in patients who respond to *KRAS* inhibitors, acquired resistance inevitably occurs within one year of medication [6, 7]. These facts aboved illustrate the importance of developing new therapeutic approaches for patients with NSCLC harboring different *KRAS*.

Cuproptosis, as a novel form of programmed cell death, occurs through direct binding of copper to the fatty acylated components of the tricarboxylic acid cycle, resulting in fatty acylated protein aggregation and subsequent iron-sulfur cluster (Fe-S cluster) protein loss, which is dependent on mitochondrial respiration [8, 9]. These processes can lead to proteotoxic stress and ultimately to cell death. Recognizing the potential of mitochondria-targeting nanomaterials in cancer therapy [10, 11], lung cancer cells are highly dependent on copper for respiration and exhibit a high expression of fatty acylated proteins. Therefore, intervening in mitochondrial respiration through copper ions emerges as a promising strategy for cancer treatment [12–15]. Copper-based metal-organic frameworks (Cu-MOFs) are emerging as promising agents in cancer therapy due to their high surface area, tunable porosity, and the biological activity of copper ions [16–18]. Moreover, Cu-MOFs can act as intrinsic therapeutic agents, demonstrating antiproliferative effects against a spectrum of cancer cell lines [19, 20]. However, exploring the cuproptosis-mediated tumor therapy of Cu-MOFs and revealing their underlying mechanism has not been reported. Therefore, the multifunctionality of Cu-MOFs confer upon them a critical advantage for cancer therapy.

**Fig. 1:**
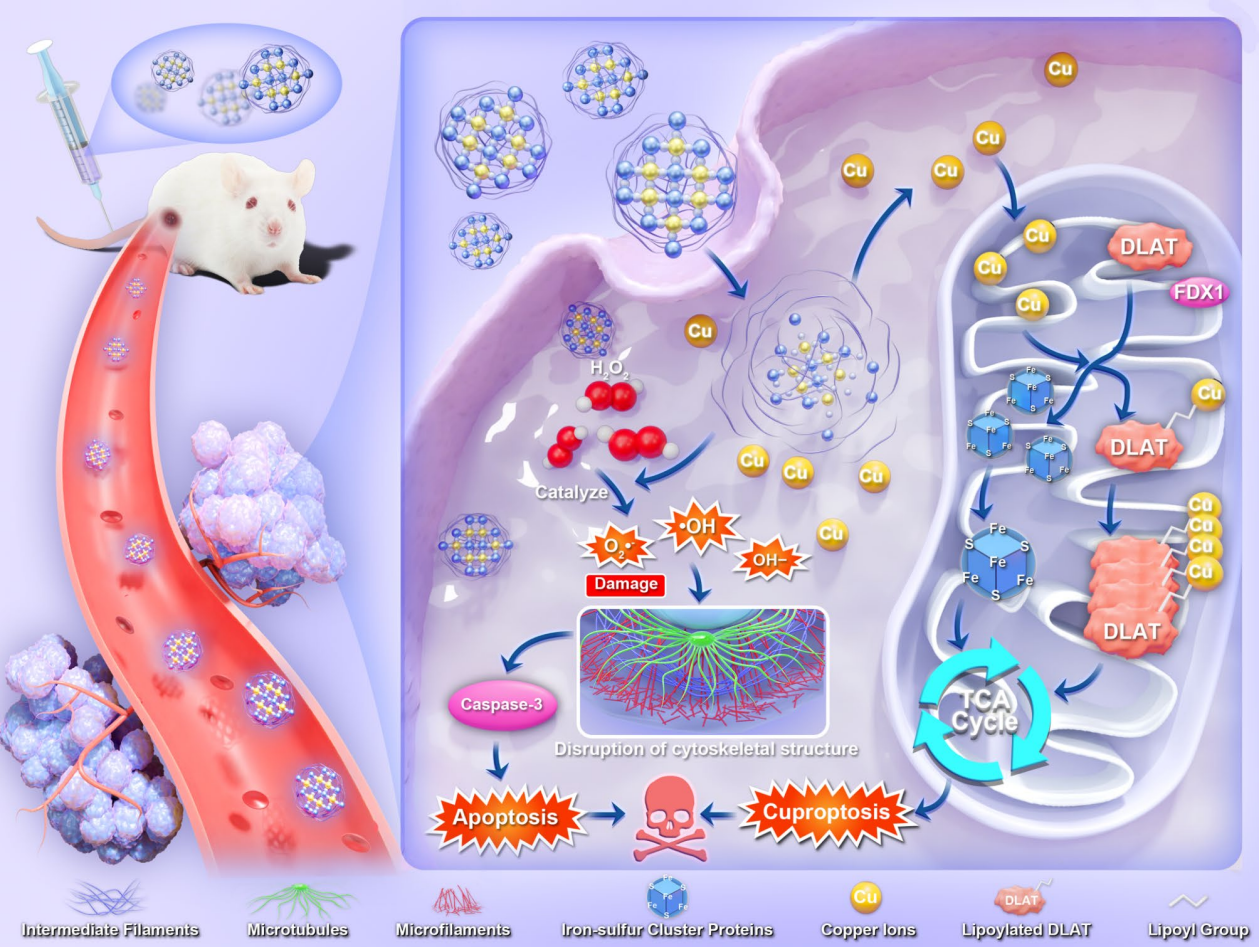
Illustration of Cu-MOF for apoptosis and cuproptosis-based cancer therapy

Herein, Cu-MOF (MOF-818) is synthesized and applied its possibility for the treatment of NSCLC. The copper ions are dissolved and released under the acidic microenvironment of lysosomes [21–23]. Given that tumor cells contain higher levels of hydrogen peroxide (H_2_O_2_), this environment promotes the Fenton reaction catalyzed by copper, leading to the generation of toxic hydroxyl radicals (•OH). The overproduction of •OH induces oxidative damage to the cytoskeleton, which further accelerates the activation of caspase-3, thereby triggering apoptosis. Importantly, we find that when copper ions bind to DLAT, a mitochondrial protein, they initiate a chain reaction that involves the critical mediator FDX1. This reaction sequence results in the loss of iron-sulfur cluster (Fe-S cluster) proteins and the aggregation of lipoylated proteins, further disrupting mitochondrial respiration and culminating in proteotoxic stress-induced cuproptosis (Fig. 1). We believe that the proposed strategy of copper-based dual apoptosis and cuproptosis-induced tumor therapy would bring new perspectives and chances for lung cancers as well as other cancers therapy.

## Results and discussion

### Synthesis and characterization of Cu-MOF

**Fig. 2:**
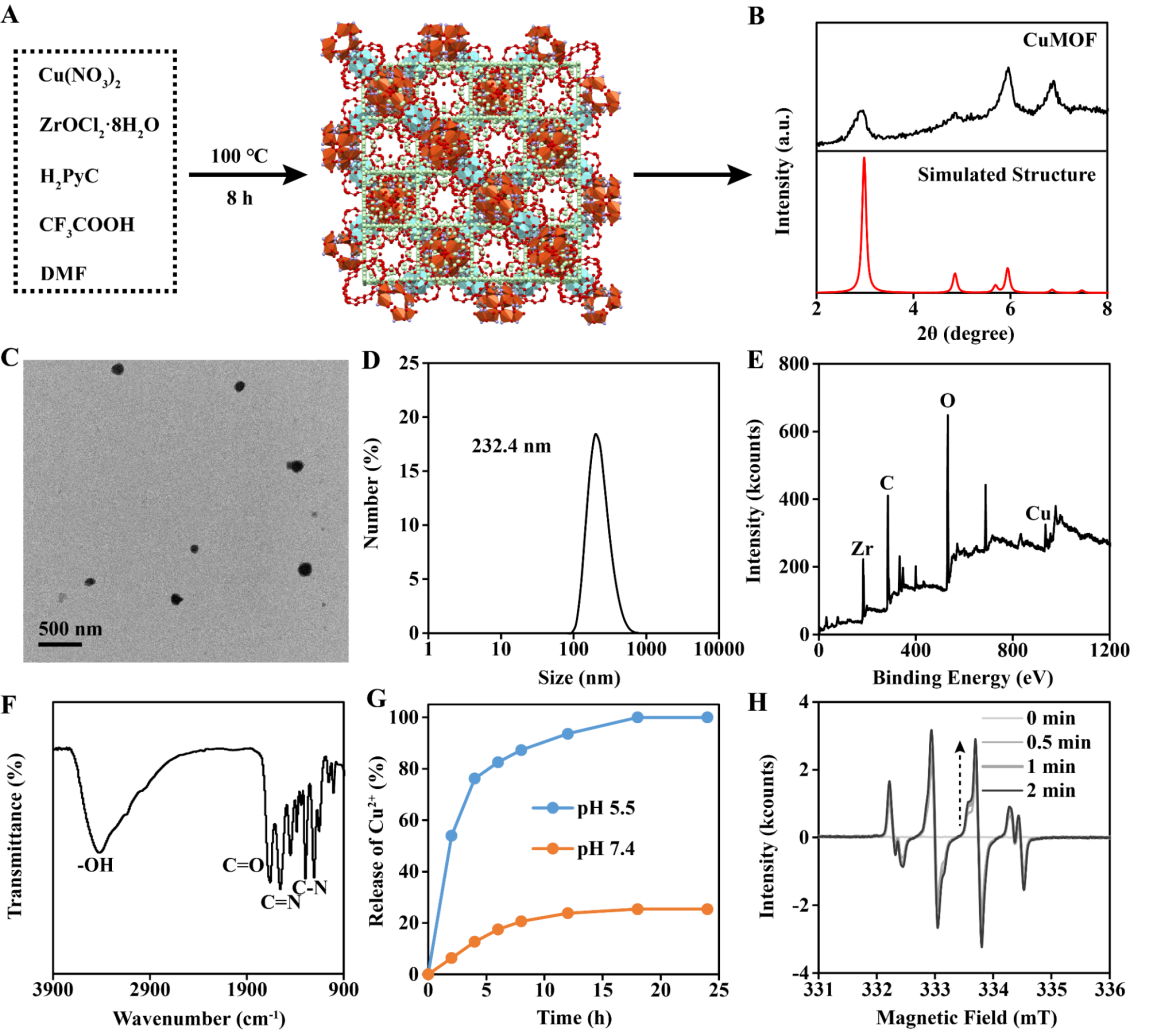
Characterization of Cu-MOF. (**A**) Synthesis progress of Cu-MOF. (**B**) XRD analysis and (**C**) TEM image of Cu-MOF. (**D**) Hydrodynamic size of Cu-MOF detected by DLS. (**E**) XPS analysis and (**F**) FTIR spectra of Cu-MOF. (**G**) Releasing analysis of Cu^2+^ from Cu-MOF. (**H**) The generation of •OH detected by ESR

The Cu-MOF was synthesized through a published method [24]. Briefly, Cu(NO_3_)_2_, ZrOCl_2_·8H_2_O, CF_3_COOH, 1 H-pyrazole-4-carboxylic acid (H_2_PyC) and dimethylformamide (DMF) were mixed. After reacting under 100 ℃ for 8 h, the Cu-MOF was obtained (Fig. 2A). The structure of Cu-MOF was MOF-818 as proved by X-ray diffraction (XRD) (Fig. 2B). Transmission electron microscopy (TEM) and dynamic light scattering (DLS) presented the size of Cu-MOF was about 200 nm (Fig. 2C-D). X-ray photoelectron spectroscopy (XPS) indicated the existence of Cu atom in Cu-MOF (Fig. 2E). Fourier transform infrared (FTIR) spectra showed the existence of H_2_PyC skeleton in Cu-MOF (Fig. 2F). In the Cu^2+^ release experiment, it was observed that Cu^2+^ almost completely released within 12 h at pH 5.5, while few Cu^2+^ was detected under pH 7.4 (Fig. 2G). Electron spin resonance (ESR) showed that Cu-MOF could effectively generate •OH under TME (100 µM H_2_O_2_ and pH 5.5), which was due to the Fenton reaction catalyzed by released Cu^2+^ (Fig. 2H).

### In vitro tumor therapeutic efficacy of Cu-MOF

**Fig. 3:**
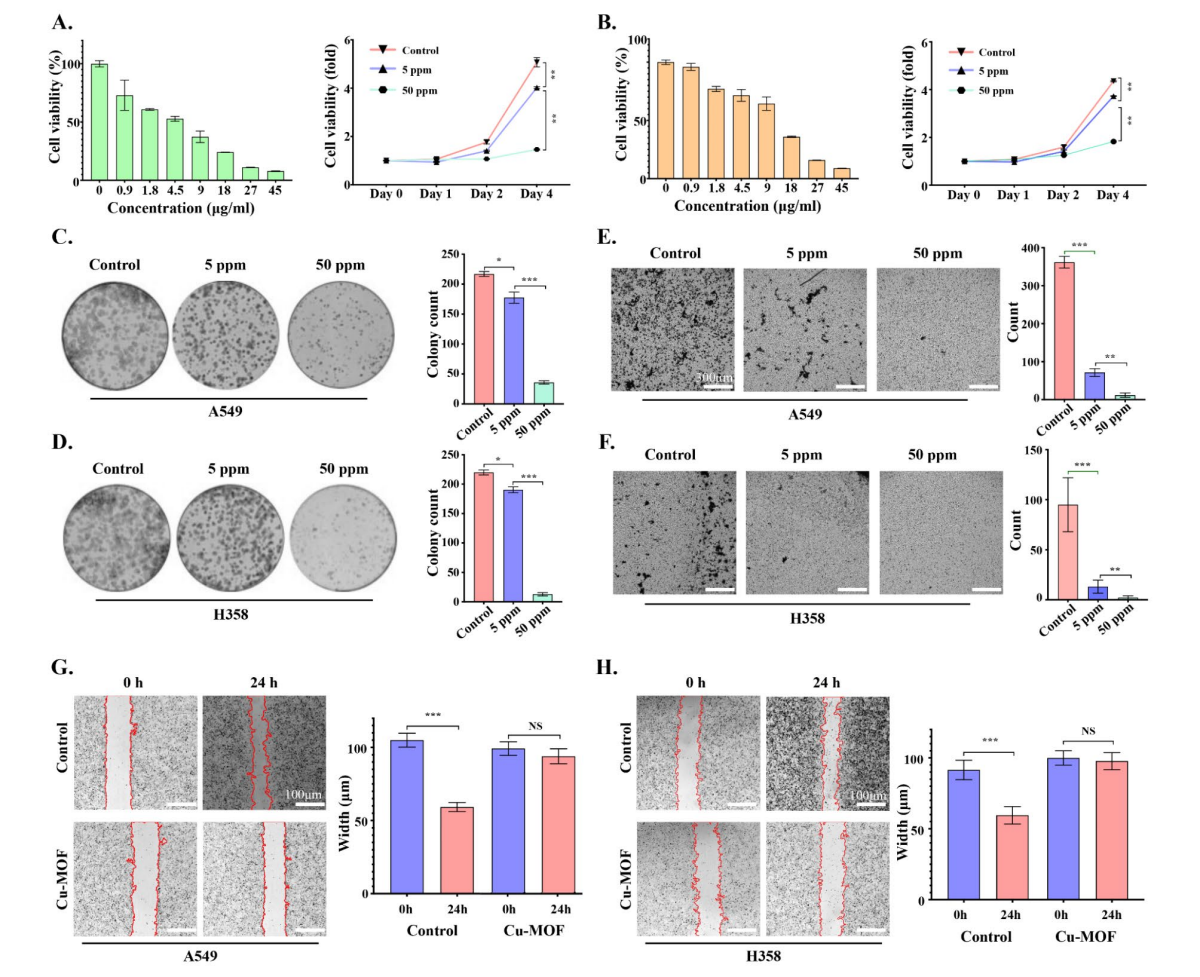
Tumor therapeutic efficacy of Cu-MOF nanoparticlesin vitro. Cell viability of A549 (**A**) and H358 (**B**) cells after intervention of Cu-MOF with various concentration gradients at 72 h and various treatments with PBS, low-dose (5 ppm), and high-dose (50 ppm) Cu-MOF after 4 days. Clonal formation of A549 (**C**) and H358 (**D**) cells treated with PBS, low-dose (5 ppm), and high-dose (50 ppm) Cu-MOF. Cell invasion behavior and numbers of A549 (**E**) and H358 (**F**) cells after different treatments were observed using the transwell system. Cell migration behavior and width of A549 (**G**) and H358 (**H**) cells after different treatments were observed. Scale bar lengths represent 300 μm in Figure E & F and 100 μm in Figure G & H. One-way analysis of variance (ANOVA) was performed: ^***^p < 0.001, ^**^p < 0.01, ^*^p < 0.05

In vitro therapy efficacy of Cu-MOF in A549, SW-1573, NCI-H1975 and NCI-H358 cells was determined by the MTT assay. As shown in Fig. 3A-B and Figure S1-2-3-4, with the increase of Cu-MOF concentration gradient, the cell viability of A549, NCI-H358, NCI-H1975 and SW1573 cell lines decreased gradually after 72 h of intervention. In addition, compared with the control group, except for SW1573, low-dose Cu-MOF (5 ppm) could reduce cell viability of the other three cell lines (A549, NCI-H1975 and NCI-H358), and the difference was statistically significant. At the same time, high doses of the Cu-MOF (50 ppm) showed a higher killing efficacy on four lung cancer cell lines on day 4 of the intervention. In concordance, the clone formation of A549 and NCI-H358 cells decreased significantly in low-dose Cu-MOF (5 ppm) group compared with control group (Fig. 3C-D). Although low doses of Cu-MOF did not significantly inhibit the clonal formation of two cell lines SW-1573 and NCI-H1975 (Figure S1E-F), high doses of Cu-MOF had superior anticancer efficacy on all four lung cancer cell lines, and there was a significant statistical difference (Fig. 3C-D and Figure S5-6-7-8). Meanwhile, the number of cell invasions were significantly reduced in the low-dose group, and a much lower ratio occurred in the high-dose group compared to the control group (Fig. 3E-F). Furthermore, cells in the control group almost crawled all over the scratched area after 24 h with a migration rate of 43.5% and 34.9% in A549 and H358, respectively, while the migration of cells treated with high-dose Cu-MOF was significantly inhibited in both group (Fig. 3G-H). These above cellular results showed effective therapeutic effects of Cu-MOF, motivating us to explore its deep anti-tumor mechanism.

### Transcriptomics analysis of Cu-MOF treated tumor cells

**Fig. 4:**
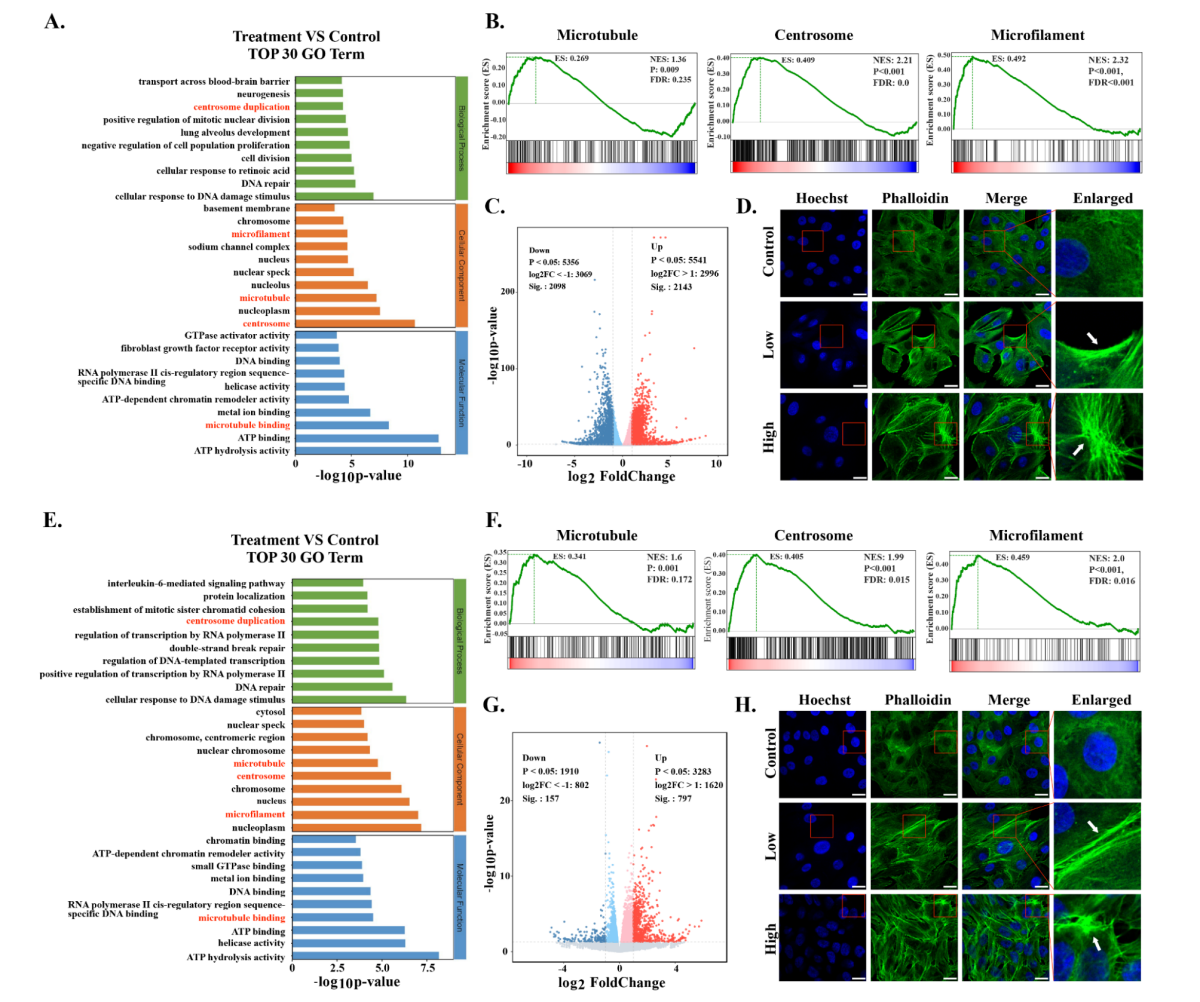
Transcriptome Sequencing Analysis after Cu-MOF Interventionin vitro. Top 30 GO term of differentially expressed genes (DEGs) between the control and high-dose (50 ppm) Cu-MOF groups in both A549 (**A**) and H358 (**E**) cells. Gene set enrichment analysis (GSEA) for microtubules, centrosome and microfilaments related Cu-MOF therapy in both A549 (B) and H358 (**F**) cells. Volcano plot based on DEGs between the control and high-dose (50 ppm) Cu-MOF groups in both A549 (**C**) and H358 (**G**) cells. Confocal images of A549 (**D**) and H358 (**H**) cell lines after incubation with PBS and high-dose (50 ppm) Cu-MOF nanoparticles. Scale bar = 20 μm

Transcriptomics analysis was carried out to explore potential anti-tumor mechanisms induced by Cu-MOF. The volcano plot illustrated significant changes of gene expression between the control group and Cu-MOF group (Fig. 4C-G). GO analysis demonstrated that the cytoskeletal structures were perturbed by Cu-MOF in both A549 and H358 cells obviously (Fig. 4A-E). Gene set enrichment analysis (GSEA) further confirmed significant differences of gene expression related to centrosome, microtubule, and microfilament between the Cu-MOF group and control group (Fig. 4B-F). To further verify the destructive effect of Cu-MOF on the cytoskeletal structures of lung cancer cells, we measured fluorescence of phalloidin and found that high-dose Cu-MOF strongly depolymerized cytoskeletal network (Fig. 4D-H). These results suggested that the cytoskeletal structures was visibly disassembled by Cu-MOF.

As a dynamic network of intermediate filaments, microtubules, and microfilaments, the cytoskeleton plays a crucial role in maintaining cellular integrity and regulating various cellular processes including apoptosis [25]. It has been reported that the disruption of the cytoskeleton triggered a cascade of events, including a decrease in mitochondrial transmembrane potential, an enhanced release of cytochrome c, which is essential for the activation of caspase-9 and the subsequent initiation of the caspase cascade [26, 27]. These events were found to initiate earlier and proceed more rapidly in cells with compromised cytoskeletal structures. Furthermore, the formation of apoptotic bodies was influenced by the state of the actin system, suggesting that the cytoskeleton not only affects the early biochemical events of apoptosis but also the later morphological changes. These results underscores the complex interplay between the cytoskeleton and apoptotic pathways, demonstrating that cytoskeletal disruption markedly accelerates caspase-3 activation, a pivotal player in the apoptotic cascade, thereby intensifying apoptotic progression [28, 29].

### Cu-MOF induce tumor cell apoptosis

**Fig. 5:**
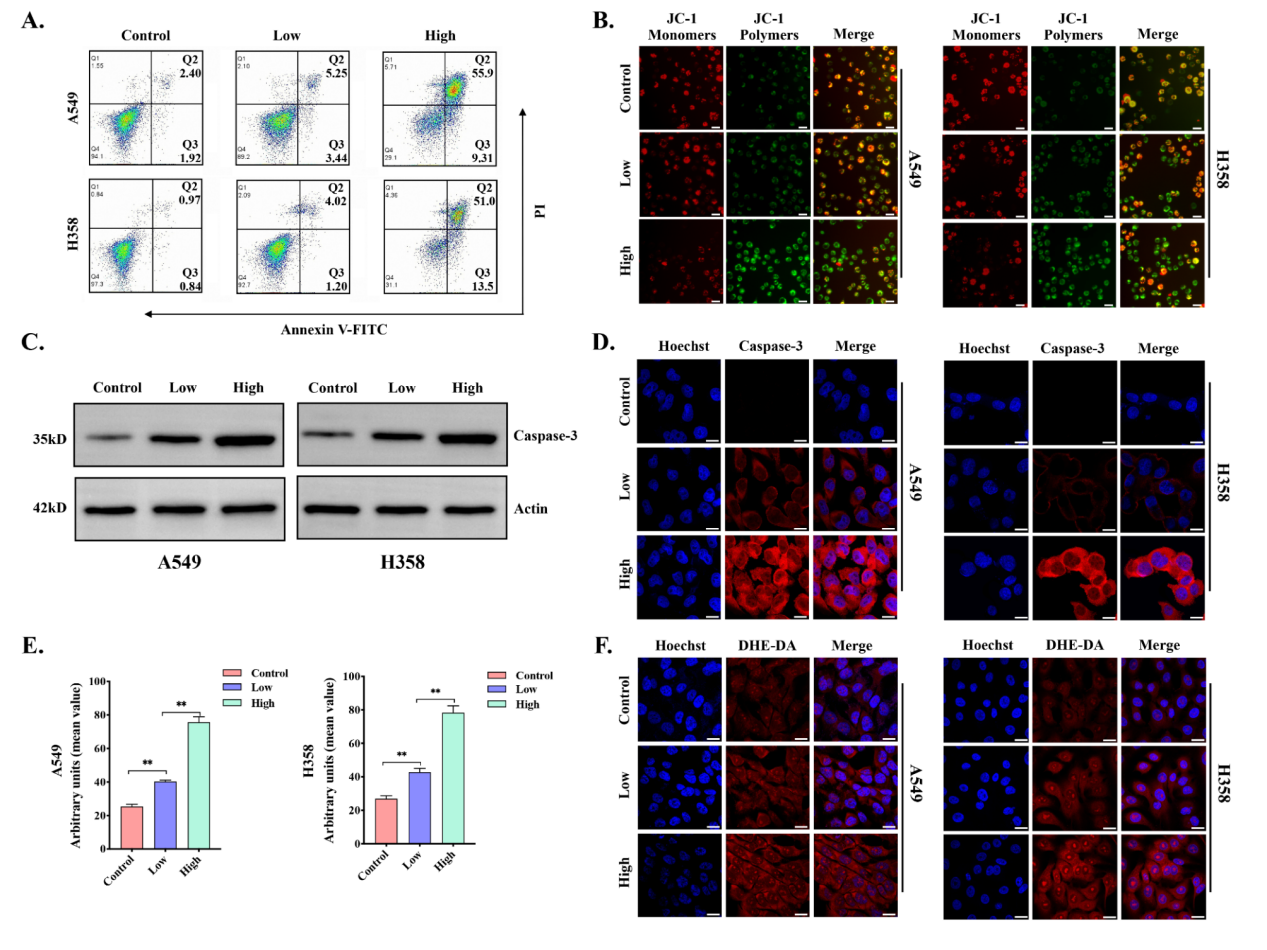
In vitro tumor apoptosis induced by Cu-MOF. (**A**) Flow cytometric analysis of A549 and H358 cells treated with PBS, low-dose (5 ppm), and high-dose (50 ppm) Cu-MOF (*n* = 3, mean ± SD). (**B**) Mitochondrial membrane potential (JC-1) fluorescence images of A549 and H358 cells after different treatments (PBS, low-dose (5 ppm), and high-dose (50 ppm) Cu-MOF). (**C**) Western blot analysis of the expression of casepase-3 in A549 and H358 cells treated with PBS, low-dose (5 ppm), and high-dose (50 ppm) Cu-MOF. (**D**) Casepase-3 fluorescence images of A549 and H358 cells after different treatments (PBS, low-dose (5 ppm), and high-dose (50 ppm) Cu-MOF). (**E**) Arbitrary units of ROS fluorescence intensity in A549 and H358 cells after different treatments (PBS, low-dose (5 ppm), and high-dose (50 ppm) Cu-MOF) (*n* = 3, mean ± SD). (**F**) Fluorescence images of A549 and H358 cells with ROS produced after different treatments (PBS, low-dose (5 ppm), and high-dose (50 ppm) Cu-MOF). Scale bars: 20 μm. One-way analysis of variance (ANOVA) was performed: ^***^p < 0.001, ^**^p < 0.01, ^*^p < 0.05

To further explore whether apoptosis occurred in lung cancer cell lines after Cu-MOF intervention, flow cytometry Annexin V-FITC and PI staining were conducted. The data indicated that the proportion of viable A549 and H358 cells were 94.1% and 97.3% in the control group, respectively. Importantly, the viability of A549 and H358 cells were further decreased to 29.1% and 31.1% in the high-dose group (Fig. 5A). At the same time, the apoptosis rate of A549 cell line after intervention ranged from 4.32 to 65.21%, and the data ranged from 1.81 to 64.5% in H358. When A549 and H358 cell lines were cultured with different doses of Cu-MOF for 24 h, the proportion of cells in each stage of the cell cycle was detected. Compared with the control group, the proportion of cells in G2/M phase was significantly increased (A549: from 12.81 to 18.32%; H358: from 12.91 to 19.41%), and the proportion of cells in G0/G1 phase was significantly decreased, and this trend became more and more obvious with the increase of concentration (Figure S9-10-11-12).

Caspase-3 is considered to be a key executive molecule in the early stage of apoptosis, and the activation of Caspase-3 leads to cellular size reduction, cell membrane blistering, chromatin aggregation, and nuclear division, which are typical morphological features of apoptotic cells. Western blot analysis and immunofluorescence confirmed that the activity of Caspase-3 increased significantly with the increase of the intervention concentration of Cu-MOF (Fig. 5C-D). In addition, the decline in mitochondrial membrane potential is a signature event in the early stage of apoptosis, which can be easily detected by the transition of JC-1 from red to green fluorescence, and our study also confirmed that the mitochondrial membrane potential decreased significantly with the increase of the concentration of Cu-MOF (Fig. 5B).

Reactive oxygen species (ROS), including the hydroxyl radical (•OH), hydrogen peroxide (H_2_O_2_), singlet oxygen (O_2_), and the superoxide radical anion (O_2_^•−^), are predominantly produced by the mitochondrial respiratory chain during intracellular redox reactions. Given that tumor cells contain higher levels of hydrogen peroxide (H_2_O_2_), this microenvironment promotes the Fenton reaction catalyzed by copper, leading to the generation of toxic hydroxyl radicals (•OH). An excessive accumulation of ROS can lead to oxidative damage across various cellular components, including nucleic acids, mitochondria, and proteins, potentially culminating in cell apoptosis [8]. To further investigate the mechanism by which Cu-MOF disrupts the cytoskeletal structure leading to apoptosis, we analyzed the levels of intracellular ROS in A549 and H358 cells by CLSM analysis (Fig. 5E-F). As the concentration of the Cu-MOF increased, DHE-DA fluorescence intensities were prominently enhanced. The high-dose group showed the highest fluorescence signal (Fig. 5E-F). These results further confirmed that the Cu-MOF had superior anticancer efficacy, which increased with the increase of Cu-MOF concentration. Under the tumor microenvironment, an adequate amount of copper ions is released, which catalyzes the conversion of H_2_O_2_ into •OH to cause damage to cellular components, particularly the cytoskeletal structure. This disruption further induces G2/M phase arrest and triggers the activation of caspase-3, ultimately leading to the induction of apoptosis in *KRAS* mutant NSCLC cells.

### Cu-MOF induce tumor cell cuproptosis

**Fig. 6:**
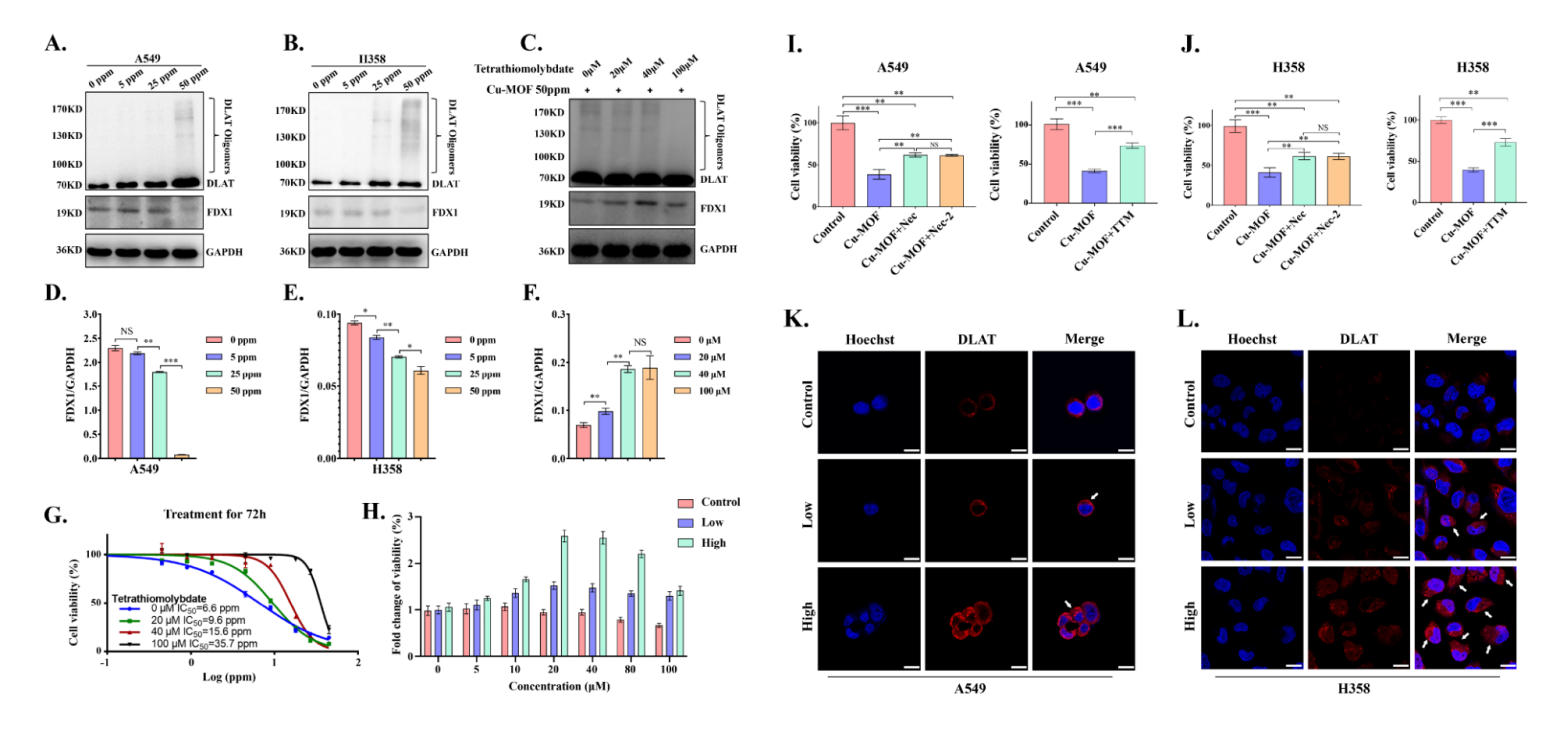
In vitro tumor cuproptosis induced by Cu-MOF. Western blot analysis of the expression of DLAT oligomerization and Fe − S cluster protein in A549 (**A**) and H358 (**B**) cells after the indicated treatments. (**C**) DLAT oligomerization and Fe − S cluster protein expression in H358 cells after co-treated with high-dose (50 ppm) Cu-MOF and cuproptosis inhibitors TTM (0, 20, 40, 100 µM). Ratio of FDX1/GAPDH expression in A549 (**D**), H358 (**E**) cells after the indicated treatments. (**F**) Ratio of FDX1/GAPDH expression in H358 cells after co-treated with high-dose (50 ppm) Cu-MOF and cuproptosis inhibitors TTM (0, 20, 40, 100 µM). (**G**) Cell viability curve of H358 cells co-treated with high-dose (50 ppm) Cu-MOF and cuproptosis inhibitors TTM (0, 20, 40, 100 µM) 72 h. (**H**) Fold change of viability of H358 cells co-treated with different treatments (PBS, low-dose (5 ppm), and high-dose (50 ppm) Cu-MOF) and cuproptosis inhibitors TTM (0, 5, 10, 20, 40, 80, 100 µM) 72 h. Cell viability of A549 (**I**) and H358 (**J**) cells co-treated with Cu-MOF and apoptosis inhibitors (Necrosulfonamide [Nec] at 10 µM and Necrostatin-2 racemate [Nec-2] at 10 µM) and cuproptosis inhibitors (Triethylenetetramine [TTM] at 30 µM) 72 h. DLAT fluorescence images of A549 (K) and H358 (**L**) cells after different treatments (PBS, low-dose (5 ppm), and high-dose (50 ppm) Cu-MOF). White arrows indicated DLAT aggregation. Scale bars: 20 μm. Student’s t-test was performed: *p < 0.05, **p < 0.01, ***p < 0.001

Cuproptosis induced by excess intracellular copper is a novel form of programmed cell death involving lipoylated protein aggregation and subsequent iron-sulfur cluster (Fe-S cluster) protein loss, which is dependent on mitochondrial respiration [30, 31]. As a mitochondrial lipidated proteins, DLAT is directly bound by copper ions to produce lipidated protein aggregation, which ultimately promotes proteotoxic stress-induced copper death. Western blotting assay and confocal images were conducted to detect DLAT aggregation. When A549 and H358 cells treated with different dose of Cu-MOF, we found that the increased Cu-MOF concentration increased the degree of DLAT aggregation by protein expression and mitochondrial fluorescence assay (Fig. 6A-B-K-L). In addition, Ferredoxin 1 (FDX1) was identified as the key gene associated with cuproptosis, which was directly bound to copper ions and lead to inhibition of the Fe-S cluster formation function. Western blot results also showed that the expression of FDX1 protein decreased gradually with the increase of Cu-MOF concentration (Fig. 6A-B-D-E).

To confirm that cuproptosis plays a role in Cu-MOF induced cell death, TTM (Tetrathiomolybdate, a copper-chelating agent) was used to evaluate the effect of copper chelation on the cytotoxicity of Cu-MOF in H358 cancer cells. The cell viability was measured after combined treatment with Cu-MOF and TTM, and then was expressed as fold change relative to that of the cells in Cu-MOF intervention alone. As shown in Fig. 6G-H, significantly greater rescuing effects, including cell viability and fold change of viability, were occurred in Cu-MOF and TTM co-treated cells, compared with Cu-MOF intervention alone. In co-culture cell lines, with the increasing concentration of TTM, the degree of DLAT aggregation decreased and the expression of FDX1 increased significantly (Fig. 6C-F), which also indicated that cuproptosis was rescued after TTM intervention in Cu-MOF-treated lung cancer cells.

To further determine the predominant mode of cell death in antitumor effect of Cu-MOF, we conducted rescue experiments on cells treated with Cu-MOF, utilizing both apoptosis inhibitors including Necrosulfonamide (i.e., Nec) and Necrostatin-2 racemate (i.e., Nec-2) and cuproptosis inhibitors of Triethylenetetramine (i.e., TTM). In line with our previous findings, Cu-MOF significantly reduced cell viability to 38.5% in A549 cells and 41.1% in H358 cells. However, upon the addition of Nec and Nec-2, there was a significant improvement in cell viability for both cell lines, which was increased to 61.8% for A549 and 61.5% for H358 with Cu-MOF + Nec, and to 61.3% for A549 and 61.2% for H358 with Cu-MOF + Nec-2 (Fig. 6I-J). Moreover, a comparison between Cu-MOF alone and the combination of Cu-MOF with TTM demonstrated a substantial increase in cell viability from 41.3 to 73.8% in the A549 cell line and from 39.8 to 73.1% in the H358 cell line. Notably, when TTM was added alone, the improvement in cell viability for both cell lines was significantly greater than that observed in groups treated with apoptosis inhibitors only (Fig. 6I-J). This indicates that cuproptosis is a more dominant form of cell death induced by Cu-MOF than apoptosis.

### In vivo the antitumor ability of Cu-MOF

**Fig. 7:**
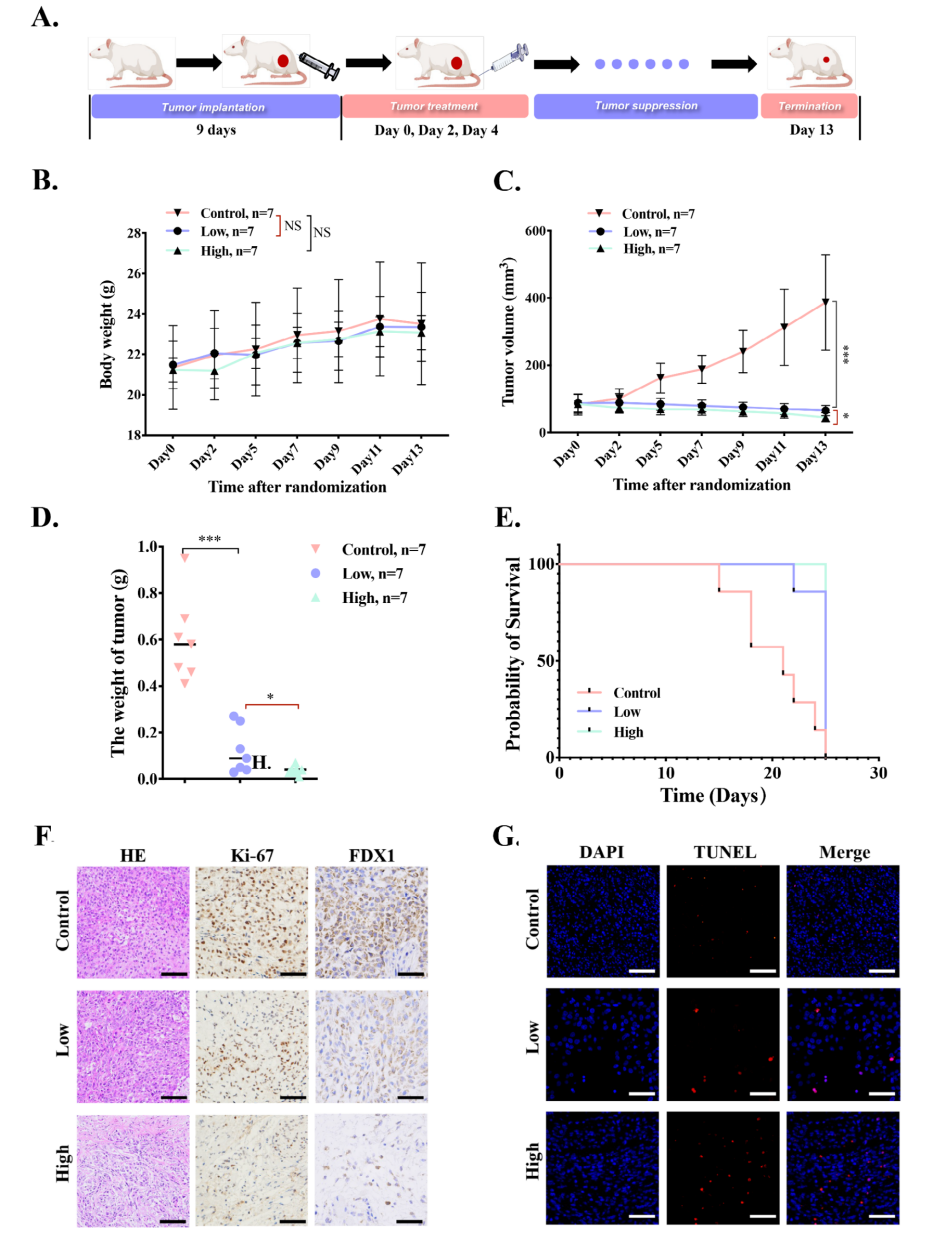
In vivo the antitumor ability of Cu-MOF. (**A**) In vivo therapeutic schedule of MOF-Cu (*n* = 7). (**B**) Body weight of BALB/c nude mice bearing A549 tumors during 13-day treatments (*n* = 7). (**C**) Tumor volume growth curves during the 13-day therapeutic period. (**D**) The weight of excised tumors on the 13th day. (**E**) Survival curves of tumor-bearing mice with different treatments during 25 days of monitoring (*n* = 7). (**F**) Representative H&E staining, immunohistochemical staining including Ki67 and FDX1 images of excised tumors among three groups after interventions. (**G**) TUNEL fluorescence images after different treatments (PBS, low-dose (0.05 mg per mouse), and high-dose (0.1 mg per mouse) Cu-MOF). Scale bars: 20 μm. Student’s t-test was performed: *p < 0.05, **p < 0.01, ***p < 0.001

To explore the in vivo anticancer efficacy of Cu-MOF on BALB/c nude mice bearing A549 tumors, we regularly monitored the body weight and tumor volume of mice for 13 days. The mice were randomly divided into three groups (*n* = 7 per group) and intratumorally injected 4 times on day 0, 2 and 4, respectively, with: (i) PBS, (ii) low-dose Cu-MOF (5 ppm), (iii) high-dose Cu-MOF (50 ppm) (Fig. 7A). Firstly, the body weight of mice increases slightly and have no significant difference among the three treatments (Fig. 7B), indicating that Cu-MOF have no system toxicity. In addition, the tumor volume in control group increases rapidly, which is four times of initial volume (Fig. 7C). Compared to the control group, low-dose Cu-MOF treatment produced 83.1% inhibition of tumor growth, at the same time, tumor growth was inhibited by 88.2% in high-dose Cu-MOF treatment group. Referring to the weight changes of tumor in three groups, tumor inhibition rates were calculated to be 82.7% and 88.8% for low-dose and high-dose group, respectively (Fig. 7D). Importantly, the tumors in high-dose Cu-MOF group did not significantly increase or even shrink within treatments. These results suggested that the Cu-MOF produced an efficient therapeutic effect. As shown in Figure S17, the tumor-suppressing effect of each group is clear in comparison. According to Kaplan-Meier survival curve, the lifespan of mice in low and high dose Cu-MOF group was significantly longer than that in control group (Fig. 7E), further evidencing the excellent antitumor efficacy of Cu-MOF.

To further examine the anti-tumor effect, H&E, and Ki-67 staining were conducted. The results of H&E staining for necrosis analysis showed that Cu-MOF especially high dose group caused the most severe damage to the tumors (Fig. 7F). Ki-67 protein, a marker of tumor proliferation, decreased significantly in the high-dose Cu-MOF group (Fig. 7F), indicating that the tumor killing ability was enhanced with the increase of dose.

To further provide a more comprehensive understanding of the cell death mechanisms induced by Cu-MOF in vivo, TUNEL assay and immunohistochemical (IHC) analysis of FDX1 were performed in our animal experiments. The TUNEL assay results indicated a pronounced increase in the signal for apoptosis in the Cu-MOF treated groups when compared to the control group, suggesting a significant induction of apoptosis (Fig. 7G). Furthermore, the IHC analysis revealed a notable decrease in the expression of FDX1, a biomarker indicative of cuproptosis, within the tumor tissues of the Cu-MOF treated mice (Fig. 7F). This finding corroborates the in vitro observations and reinforces the role of Cu-MOF in inducing cuproptosis and apoptosis.

This verified that Cu-MOF treatment laid a foundation for implementing strategies to regulate cell death to achieve better antitumor treatment results. To ascertain the biocompatibility of Cu-MOF nanoparticles, histological examination of the major organs of mice after the intervention showed that no significant alterations were observed (Figure S13). In addition, blood samples were also collected when mice were sacrificed. The results of blood routine, liver and kidney function indexes also exhibit no overt change in all group (Figure S14). Even though white blood cells and lymphocytes tend to rise as the drug concentration increases, they are still within the normal range (the dashed line is the normal range) (Figure S14A-E). The results above indicated the high biocompatibility and no appreciable in vivo toxicity of Cu-MOF.

The novel therapeutic strategy utilizing Cu-MOF for lung cancer treatment presents several compelling advantages over conventional treatments such as chemotherapy, immunotherapy, and radiotherapy. Firstly, the targeted release of copper ions in the acidic microenvironment of lysosomes allows for a more precise attack on cancer cells, minimizing damage to healthy tissues. Secondly, Cu-MOF offer a dual mechanism of action, inducing both apoptosis and cuproptosis, potentially providing a more potent therapeutic effect. This approach is particularly promising for KRAS-mutated NSCLC, a subset of patients who often exhibit resistance to immunotherapies and develop resistance to targeted therapies within a year. Additionally, Cu-MOF have demonstrated good biocompatibility and low systemic toxicity in vivo, suggesting a favorable safety profile. The innovative use of Cu-MOF as a therapeutic agent also presents a new avenue for overcoming tumor resistance to existing treatments. Furthermore, the potential for broad applicability to other cancers suggests that this strategy could revolutionize cancer therapy beyond the scope of lung cancer. These benefits highlight Cu-MOF as a promising candidate for advancing cancer treatment, warranting further exploration and development.

## Conclusion

In summary, the Cu-MOF was synthesized for treating NSCLC by dual effects of apoptosis and cuproptosis. Specifically, the acidic microenvironment of lysosomes triggered the release of an adequate amount of copper ions from Cu-MOF. The released copper ions then reacts with H_2_O_2_, generating a significant quantity of ROS, which severely disrupts cellular components, especially the cytoskeleton. This disruption further induces G2/M phase arrest and triggers the activation of caspase-3, ultimately leading to the induction of apoptosis. Simultaneously, with the mediation of the key regulatory factor FDX1, copper ions binds to the mitochondrial protein DLAT, causing aggregation of DLAT and loss of Fe-S cluster proteins, which interferes with mitochondrial respiration and subsequently triggers cuproptosis. With both apoptosis and cuproptosis induced by Cu-MOF in *KRAS* mutant NSCLC cells, an effective anti-tumor effect in vivo was observed, demonstrating its feasibility for lung cancer therapy. The revealed cuproptosis of Cu-MOF provides new insight for the biological effects of Cu-based nanomaterials and also expands the additional mechanisms of Cu-based MOF in cancer therapy.

## Electronic supplementary material

Below is the link to the electronic supplementary material.

Supplementary Material 1

## Data Availability

No datasets were generated or analysed during the current study.
