# Supplementary material for: Apoptosis and cuproptosis Co-activated Copper-based metal-organic frameworks for cancer therapy
Source: J Nanobiotechnology. 2024 Sep 6;22:546. doi: 10.1186/s12951-024-02828-3 (PMC11378619; doi:10.1186/s12951-024-02828-3)
Supplement: Supplementary file 1 — Supplementary Material 1 [file 12951_2024_2828_MOESM1_ESM.docx]

Supporting Information

Apoptosis and Cuproptosis Co-Activated Copper-based Metal-organic Frameworks for Cancer Therapy

Kun Li^#^, Leilei Wu^#^, Han Wang, Zi Fu, Jiani Gao, Xiucheng Liu, Yongfei Fan, Xichun Qin, Dalong Ni*, Jing Wang* and Dong Xie*

Dr. K. Li, Dr. J. Gao, Dr. X. Liu, Dr. Y. Fan, Dr. X. Qin, Prof. D. Xie

Department of Thoracic Surgery, Shanghai Pulmonary Hospital, School of Medicine, Tongji

University, Shanghai 200433 (P. R. China)
E-mail: [xiedong@tongji.edu.cn](mailto:xiedong@tongji.edu.cn)

Dr. L. Wu

Department of Thoracic Surgery, Zhejiang Cancer Hospital, Hangzhou Institute of Medicine (HIM), Chinese Academy of Sciences, No.1 East Banshan Road, Hangzhou, 310005, PR China.

Dr. H. Wang, Z. Fu, Prof. D. Ni

Department of Orthopaedics, Shanghai Key Laboratory for Prevention and Treatment of Bone and Joint Diseases, Shanghai Institute of Traumatology and Orthopaedics, Ruijin Hospital, Shanghai Jiao Tong University School of Medicine, Shanghai 200025 (P. R. China)

Email: [ndl12353@rjh.com.cn](mailto:ndl12353@rjh.com.cn)

Prof. D. Ni

Suzhou Institute of Biomedical Engineering and Technology, Chinese Academy of Science, Suzhou 215163 (P. R. China)

Dr. J. Wang

Department of Radiology, Shanghai Fourth People's Hospital, School of Medicine, Tongji University, Shanghai, 200434 (P. R. China)

Email: [jingwang1114@tongji.edu.cn](mailto:jingwang1114@tongji.edu.cn)

^#^Kun Li and Leilei Wu contributed equally to this work.

Keywords: Cuproptosis, Apoptosis, Cytoskeletal structure, Metal-organic frameworks, nanomedicine

Experimental Section

**Reagents**

Cu(NO_3_)_2_, ZrOCl_2_·8H_2_O, CF_3_COOH, 1H-pyrazole-4-carboxylic acid (H_2_PyC), and dimethylformamide (DMF) were obtained from Macklin. Phosphate buffered solution (PBS), RPMI 1640, Dulbecco's Modified Eagle Medium (DMEM) High Glucose, Penicillin-Streptomycin and fetal bovine serum (FBS) were purchased from Gibco. Tetrathiomolybdate (TTM), Necrosulfonamide (Nec), and Necrostatin-2 racemate (Nec-2) were purchased from TargetMol Co. Ltd., USA.

**Synthesis of Cu-MOF**

For the synthesise of Cu-MOF, 1 mmol Cu(NO_3_)_2_, 85 mg ZrOCl_2_·8H_2_O, 240 μL CF_3_COOH, 65 mg H_2_PyC and 35 mL DMF were mixed in 100 mL reaction boiler with continuous stirring for 30 min. Then this boiler was heated under 100 ℃ for 8 hours. After reaction, Cu-MOF was collected and washed with water three times. Finally, the Cu-MOF was suspended in water and stored at room temperature.

**Experimental apparatus**

Transmission electron microscope (TEM) graph was carried out by FEI Talos F200X. X-ray diffraction (XRD) was measured by Rigaku D/MAX-2250 V. X-ray photoelectron spectroscopy (XPS) was measured by Thermo Fisher Scientific ESCALAB 250XI. Dynamic light scattering was measured by Malvern Zetasizer Nano S. The content of Cu was measured by inductively coupled plasma optical emission spectrometry (ICP-OES, Agilent 725). Electron spin resonance (ESR) was detected by Bruker Magnettech ESR5000. Fourier transform infrared (FTIR) spectra were acquired on a Thermo Fisher Scientific Nicolet 6700.

**Cell lines**

The lung adenocarcinoma cell lines A549, NCI-H1975, SW1573, and NCI-H358 were obtained from American Type Culture Collection (ATCC) (Manassas, VA, USA). The A549, NCI-H1975, and NCI-H358 cells were cultured in RPMI-1640 medium (WISENT Inc., Nanjing, China) containing 100 units/mL penicillin, 100 μg/mL streptomycin and 10% fetal bovine serum (Excell Bio Inc., Taicang, China). The SW1573 cell line was cultured in DMEM medium (WISENT Inc., Nanjing, China) containing 100 units/mL penicillin, 100 μg/mL streptomycin and 10% fetal bovine serum (Excell Bio Inc., Taicang, China).

**Cell Viability Measurement**

Lung adenocarcinoma cells were seeded into 96-well plates (3000/100 μL). These cell lines were respectively treated with various concentration gradients of Cu-MOF (0 μg/ml, 0.9 μg/ml, 1.8 μg/ml, 4.5 μg/ml, 9 μg/ml, 18 μg/ml, 27 μg/ml, 45 μg/ml) for 72 hours and different concentrations of Cu-MOF (PBS, 5 ppm, 50 ppm) for four days. Cell viability was determined using a CCK8 assay (Cell Counting Kit-8, Targetmol Co. Ltd., Shanghai, China) following the manufacturer’s protocol.

**Colony Formation**

The cells were seeded into 12-well plates (1000 cells/well). Then medium changes were performed every other day, as well as medication changes. After 14–21 days of culture, the colonies were fixed with 4% paraformaldehyde and stained with 0.5% crystal violet (Yeasen Biotech Co., Ltd., Shanghai, China). The number of colonies was then counted.

**Apoptosis Detection**

Cells were incubated in 24-well plates and exposed to different concentrations of Cu-MOF (Control, 5 ppm, 50 ppm) for 72 hours. Subsequently, cells were stained with Annexin V/ propidium iodide (PI) (Elabscience, Wuhan, China). The staining was performed according to the manufacturer’s instructions. After staining, cell apoptosis was analyzed using a BD FACSCanto II flow cytometer (BD BioScience, San Jose, CA, USA).

**Cell Cycle Analysis**

After treatment with different concentrations of Cu-MOF (Control, 5 ppm, 50 ppm) for 72 hours, lung adenocarcinoma cells were harvested, washed with cold PBS, permeabilized using 70% ethanol overnight at −20 °C, and then incubated with 300 μL PI/RNase staining buffer (Elabscience, Wuhan, China) at room temperature for 15 min followed by flow cytometric analysis. Data was analyzed using ModFit LT software.

**Western Blotting**

Cells were collected, washed with cold PBS, and lysed with lysis buffer (100 mM Tris-HCl, pH 6.8, 4% SDS, 20% glycerol) on ice for 30 min. Protein concentrations were determined using Pierce™ BCA Protein Assay Kit (Thermo fisher Scientific, #23227). Primary antibodies including cleaved DLAT, GAPDH, Caspase-3 and FDX1, were from Proteintech (ProteintechGroup Inc., Chicago, USA).

**Dihydroethidium (DHE) Probe Assay**

The final concentration of DHE (Aladdin, Shanghai, China) was configured with a free medium at 10 μM. The old medium in the petri dishes was blotted out and fresh DHE was added to the solution. After the incubation for 30 min away from light, the DHE solution was aspirated and the cells was washed with a fresh solution.

**Phalloidin Staining**

The cells were washed three times with PBS and fixed with 3.75% formaldehyde in PBS solution on ice for 15 min. After that, the cells were permeabilized with PBS containing 0.5% Triton X-100 for 10 min at room temperature and washed three times with PBS. Then, we diluted 1-5 μL of phalloidin (Aladdin, Shanghai, China) with 200 μL of PBS, and added them to one coverslip, which was further incubated at room temperature for 20 min and stained. Finally, the cells were washed with PBS 2-3 times.

**Reduced Glutathione (GSH) Assay Kit**

The cell precipitates were collected and washed twice with PBS, which were resuspended in PBS and centrifuged at 600 g for 10 min. Then, the cells were resuspended after adding reagents according to the product instructions (Aladdin, Shanghai, China) and freeze-thawed 3 times repeatedly in liquid nitrogen and 37℃ water bath. The precipitate was collected by centrifugation at 8000 g for 10 minutes.

**mRNA sequence**

Total RNA was extracted using the TRIzol reagent (Invitrogen, CA, USA) according to the manufacturer’s protocol. RNA purity and quantification were evaluated using the NanoDrop 2000 spectrophotometer (Thermo Scientific, USA). RNA integrity was assessed using the Agilent 2100 Bioanalyzer (Agilent Technologies, Santa Clara, CA, USA). Then the libraries were constructed using VAHTS Universal V6 RNA-seq Library Prep Kit according to the manufacturer’s instructions.

The libraries were sequenced on a llumina Novaseq 6000 platform and 150 bp paired-end reads were generated. About 50M raw reads for each sample were generated. Raw reads of fastq format were firstly processed using fastp and the low-quality reads were removed to obtain the clean reads. Then about 40M clean reads for each sample were retained for subsequent analyses. Finally, the clean reads were mapped to the reference genome using HISAT2.

***In vivo* Animal Experiments**

**Tumor formation experiment**

BALB/C nude mice (6 weeks old) were purchased from Shanghai Laboratory Animal Center (Shanghai, China) and fed in the animal house of the Tongji University. A549 cells (5 × 10^6^) in 100 μL serum-free culture medium were inoculated subcutaneously into the right flank of each mouse. When the tumors were measurable, twenty-one mice were randomly divided into control and treatment groups. The mice in treatment groups were injected with Cu-MOF (on alternate days) via the tail vein. The control group received 100 μL PBS. Tumor size and body weight were measured every other day. Tumor volume = 4π/3× (width/2)^2^ × (length/2). At the end of the treatment, mice were euthanized. The tumor was dissected, sliced, and stained by H&E and Ki67, for histopathological analysis. In addition, the blood samples and the organs of heart, liver, spleen, lung and kidney were collected for biosafety evaluation.

**Survival experiment**

Twenty-one mice BALB/C nude were established as A549-CDX models for survival experiments. Tumor size was monitored every three days, and mice were euthanized and survival time was recorded if the following conditions occurred: 1) until the length or width of the tumor exceeded 16×16 or 17×15 mm; 2) body weight loss was more than 20%; 3) mice were severely dehydrated and skin lost its luster. If all mice in the control group were executed due to the above conditions during the observation period, then the mice in the treatment group were also euthanized, the corresponding time was recorded, and the survival curves were plotted.

All animal studies have been approved by the Review Board and Ethics Committee of Shanghai Pulmonary Hospital (ID: K22-390).

**TUNEL staining**

Cell passaging was performed on tumor tissue after dewaxing and hydration, followed by TUNEL staining (TUNEL BrightRed Apoptosis Detection Kit, Vazyme, A113-03), and sample washing after staining.

**Hematoxylin-eosin (HE) and immunohistochemical staining**

CDX tumor tissues, heart, lung, spleen, kidney, and liver were fixed with 4% paraformaldehyde, embedded in paraffin, cut into sections (4 μm), and subjected to HE staining. The sections were observed under a microscope to assess histopathological changes in the cortex. Staining of paraffin-embedded CDX tumor tissues was performed with an EnVision system according to the manufacturer’s instructions (Glostrup, Dako, Denmark). A recombinant anti-Ki67 antibody (1:1000; ab279653; Abcam Co. Ltd., UK) and FDX1 antibody (1:100; ProteintechGroup Inc., Chicago, USA) were used as the primary antibodies. Antibodies in the Dako REAL EnVision Detection System were used as secondary antibodies.

**Statistical Analysis**

The measurement data were expressed as the mean ± standard deviation (SD). Statistical analysis was performed by two-tailed Student’s t-test for comparison between two groups and one-way analysis of variance (ANOVA) followed by Turkey’s post-test for comparison of three or more groups. **p* < 0.05, ***p* < 0.01, ****p* < 0.001.

**
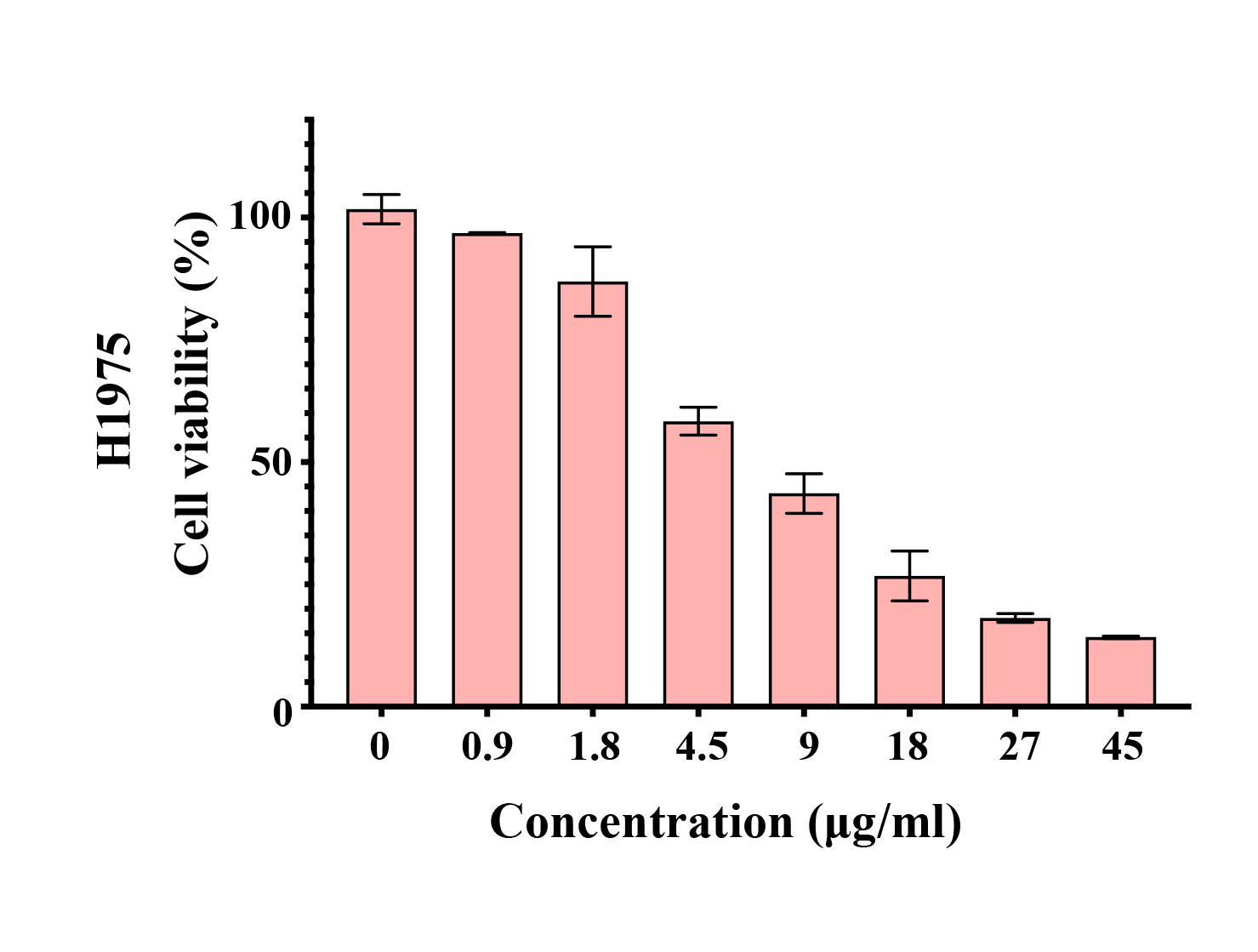
**

**Figure S1.** Cell viability of H1975 cells after intervention of Cu-MOF with various concentration gradients (0 μg/ml, 0.9 μg/ml, 1.8 μg/ml, 4.5 μg/ml, 9 μg/ml, 18 μg/ml, 27 μg/ml, 45 μg/ml) for 72 hours.


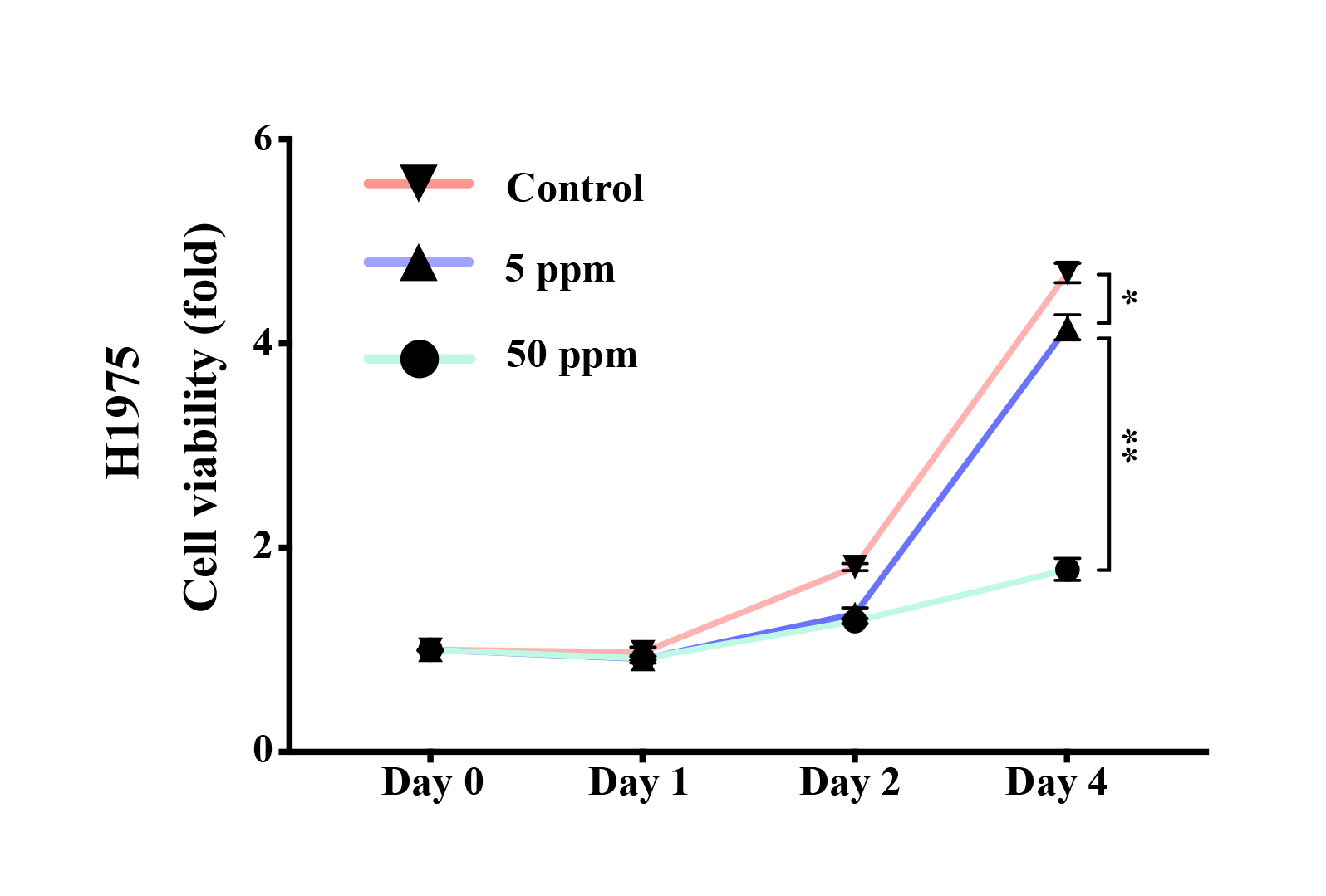


**Figure S2.** Cell viability of H1975 cells after intervention of various treatments with PBS, low-dose (5 ppm), and high-dose (50 ppm) Cu-MOF for four days. One-way analysis of variance (ANOVA) was performed: ^**^*p* < 0.01, ^*^*p* < 0.05.

**
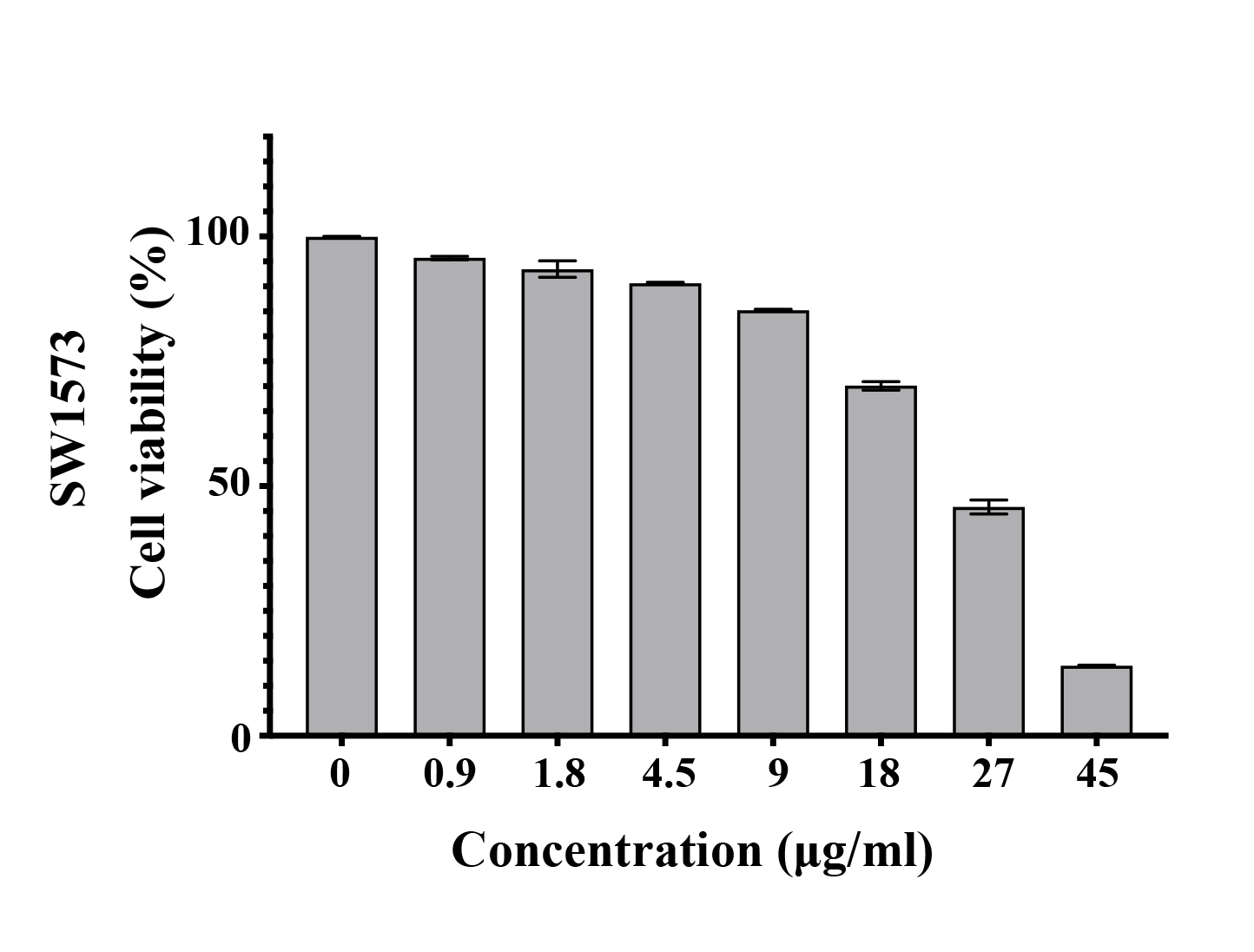
**

**Figure S3.** Cell viability of SW1573 cells after intervention of Cu-MOF with various concentration gradients (0 μg/ml, 0.9 μg/ml, 1.8 μg/ml, 4.5 μg/ml, 9 μg/ml, 18 μg/ml, 27 μg/ml, 45 μg/ml) for 72 hours.

**
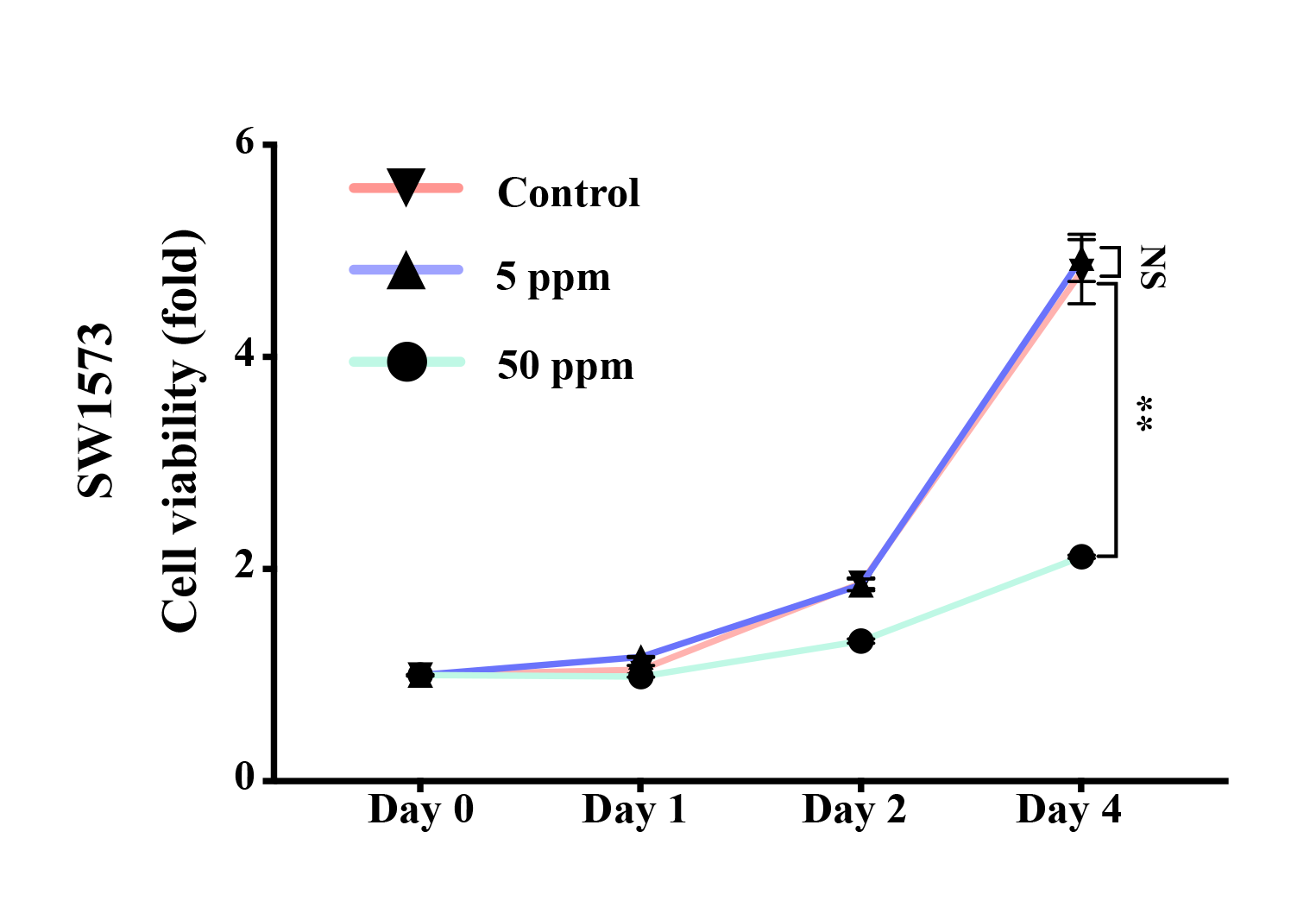
**

**Figure S4.** Cell viability of SW1573 cells after intervention of various treatments with PBS, low-dose (5 ppm), and high-dose (50 ppm) Cu-MOF for four days. One-way analysis of variance (ANOVA) was performed: ^**^*p* < 0.01. NS: no significance.

**
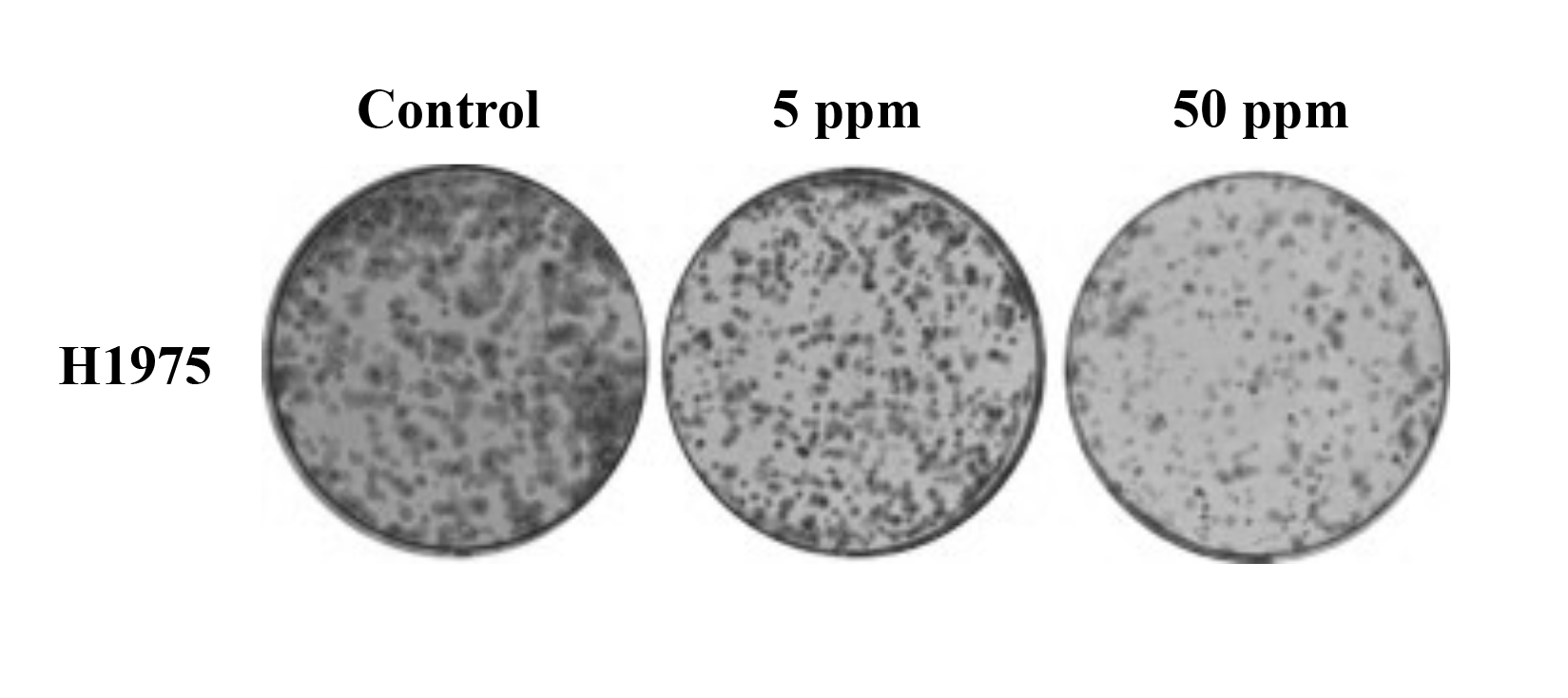
**

**Figure S5.** Clonal formation of H1975 cells treated with PBS, low-dose (5 ppm), and high-dose (50 ppm) Cu-MOF.

**
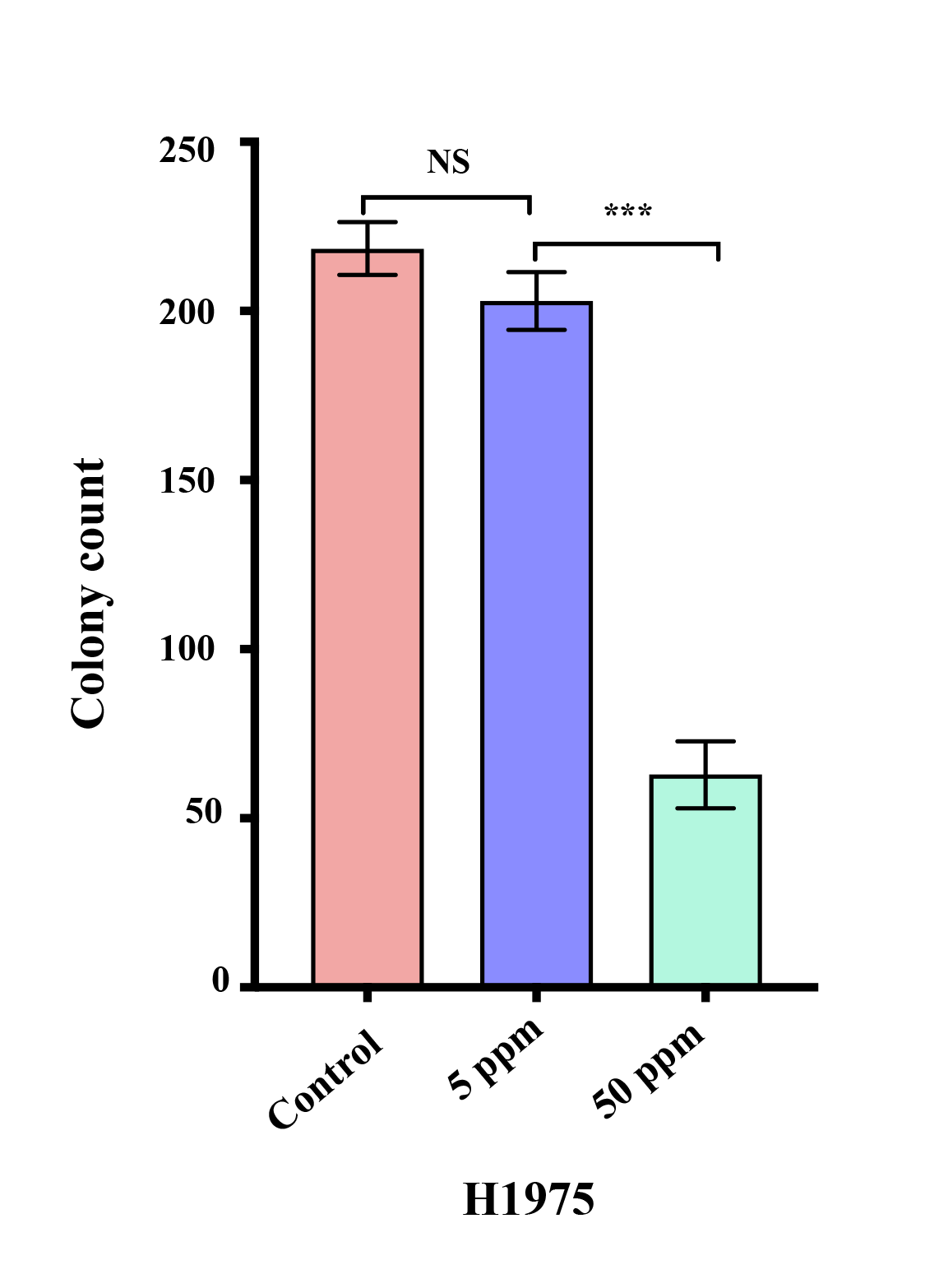
**

**Figure S6.** Colony count of H1975 cells treated with PBS, low-dose (5 ppm), and high-dose (50 ppm) Cu-MOF. One-way analysis of variance (ANOVA) was performed: ^***^*p* < 0.001. NS: no significance.

**
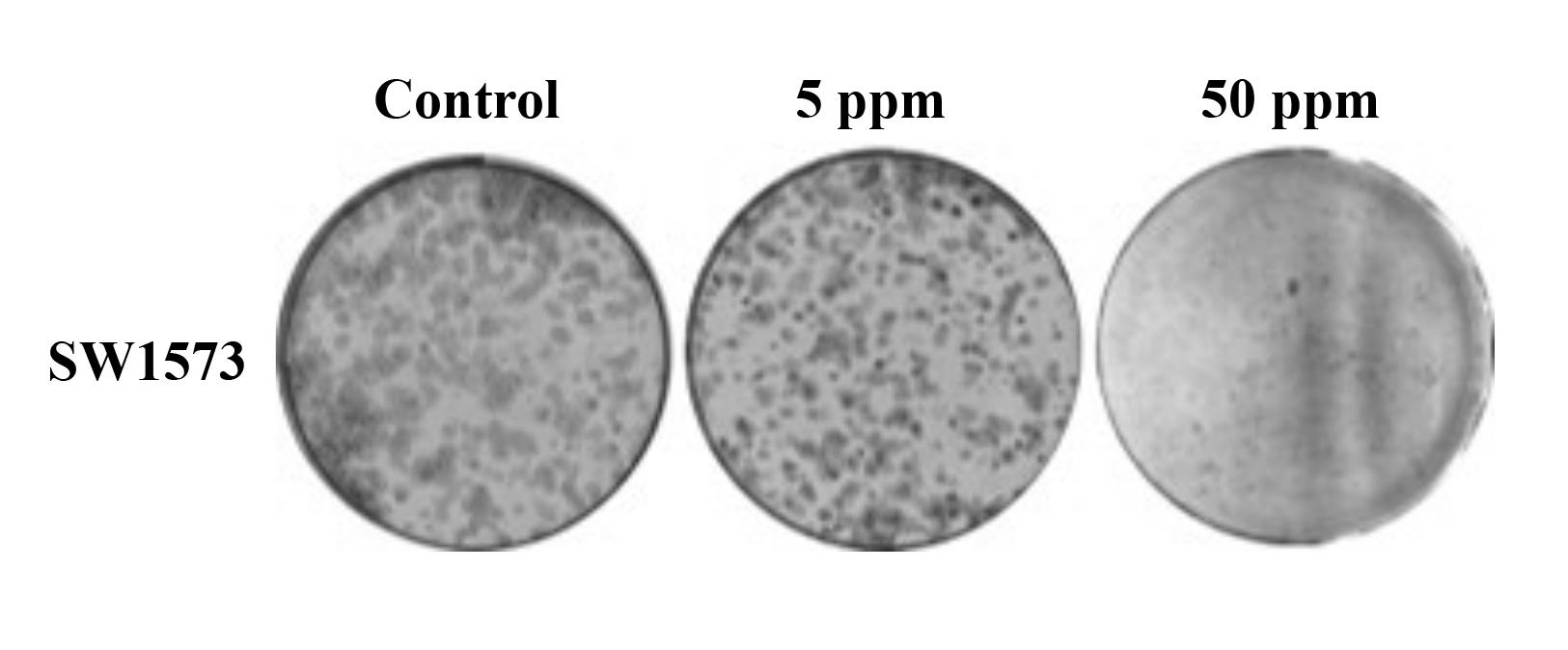
**

**Figure S7.** Clonal formation of SW1573 cells treated with PBS, low-dose (5 ppm), and high-dose (50 ppm) Cu-MOF.

**
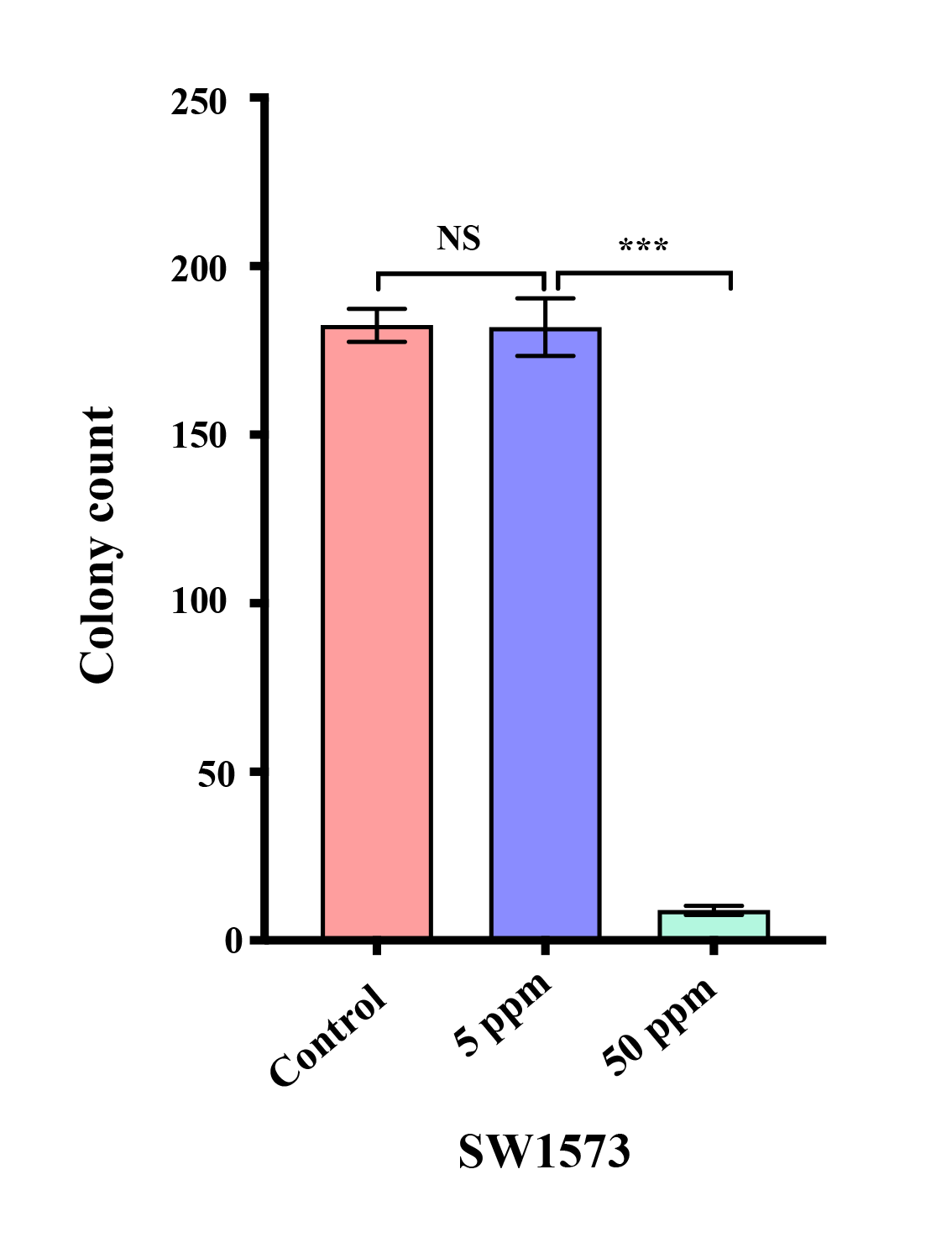
**

**Figure S8.** Colony count of SW1573 cells treated with PBS, low-dose (5 ppm), and high-dose (50 ppm) Cu-MOF. One-way analysis of variance (ANOVA) was performed: ^***^*p* < 0.001. NS: no significance.


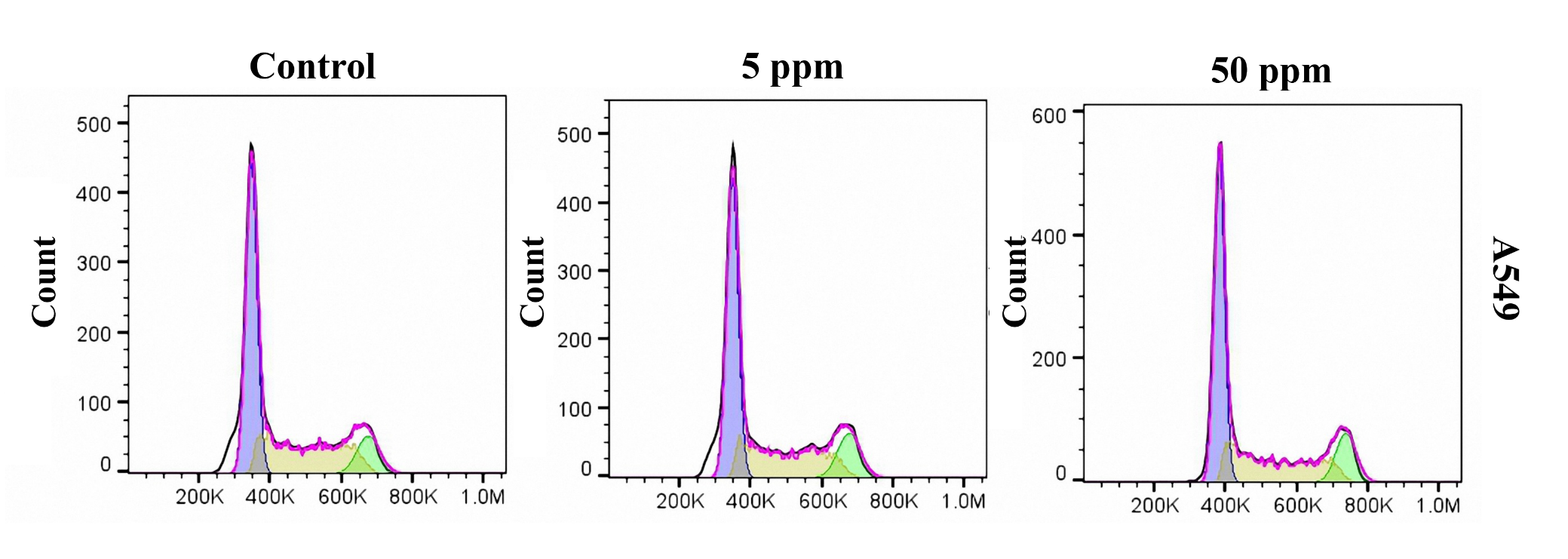


**Figure S9.** Cell cycle analysis of A549 cells treated with PBS, low-dose (5 ppm), and high-dose (50 ppm) Cu-MOF.


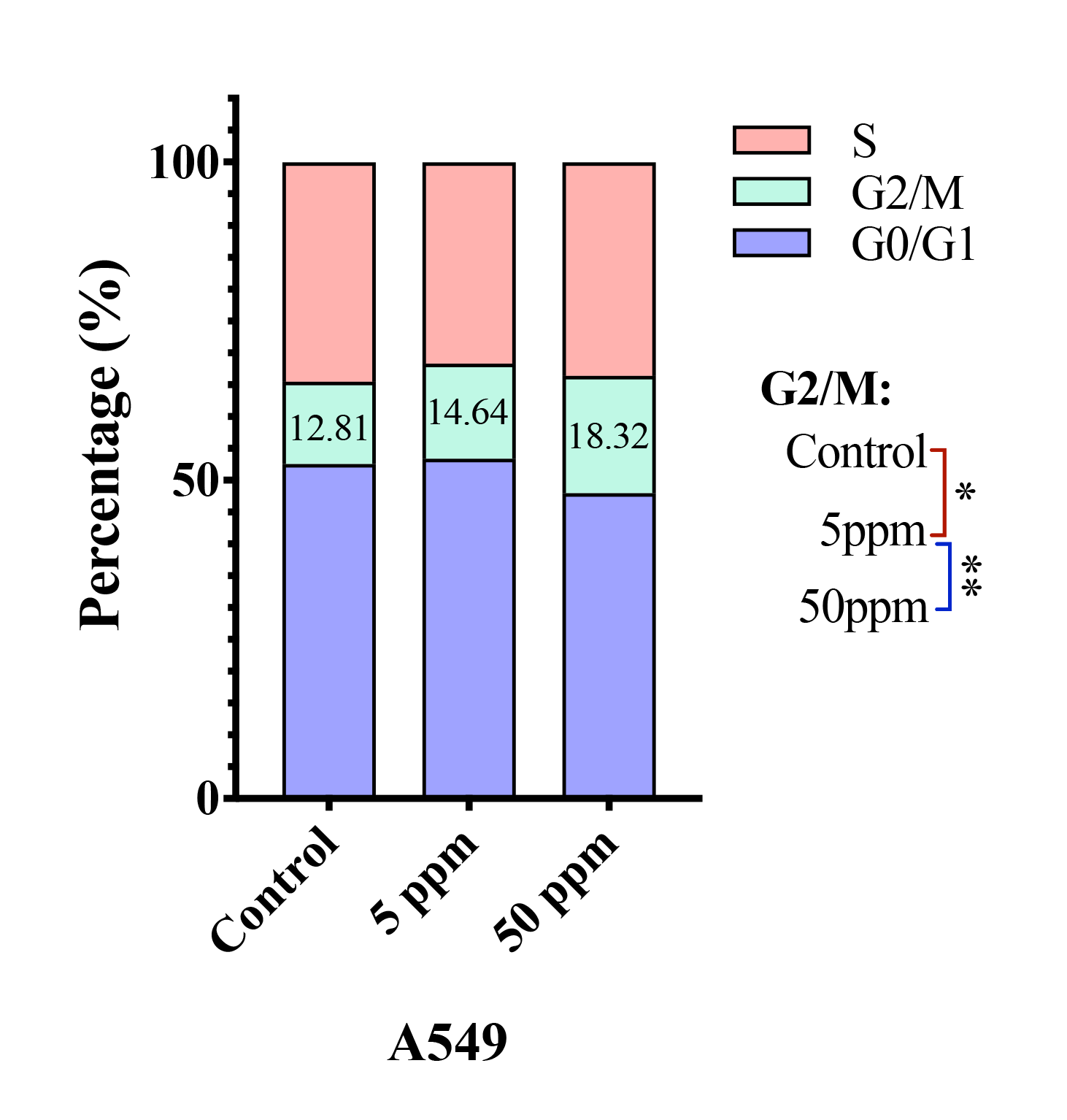


**Figure S10.** The proportion of cell division cycle of A549 cells treated with PBS, low-dose (5 ppm), and high-dose (50 ppm) Cu-MOF (n=3, mean ± SD). One-way analysis of variance (ANOVA) was performed: ^**^*p* < 0.01, ^*^*p* < 0.05.

**
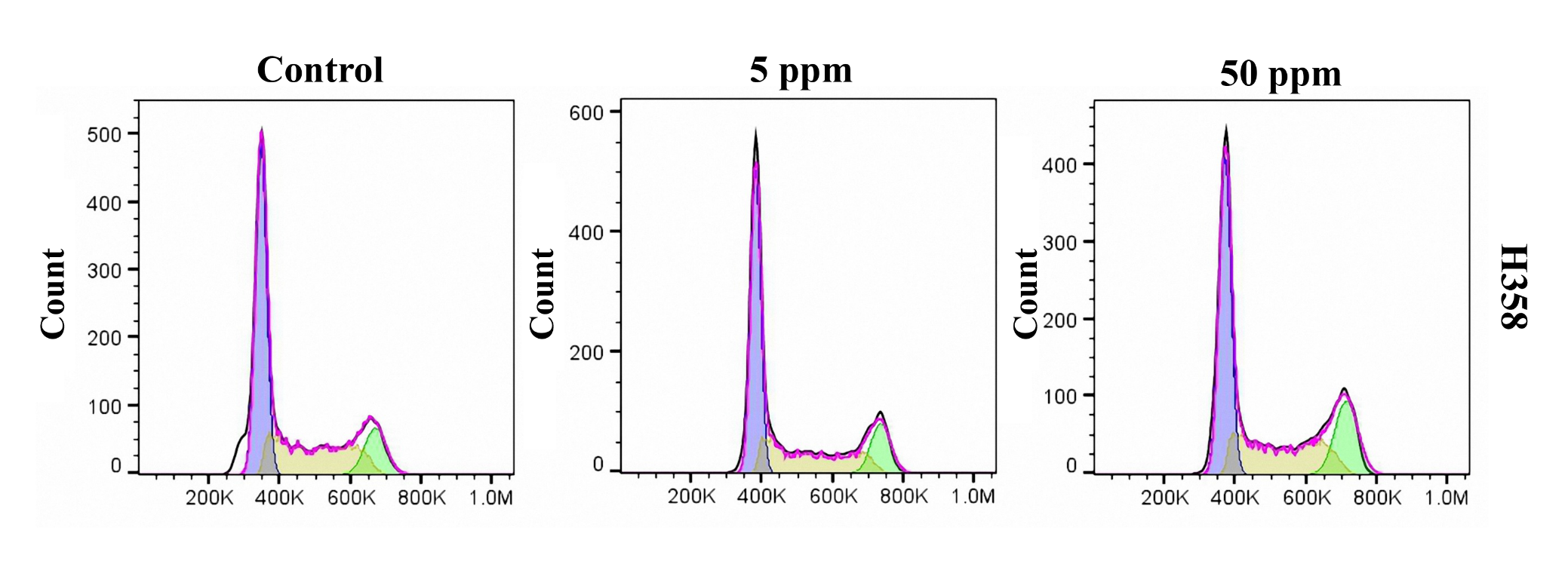
**

**Figure S11.** Cell cycle analysis of H358 cells treated with PBS, low-dose (5 ppm), and high-dose (50 ppm) Cu-MOF.

**
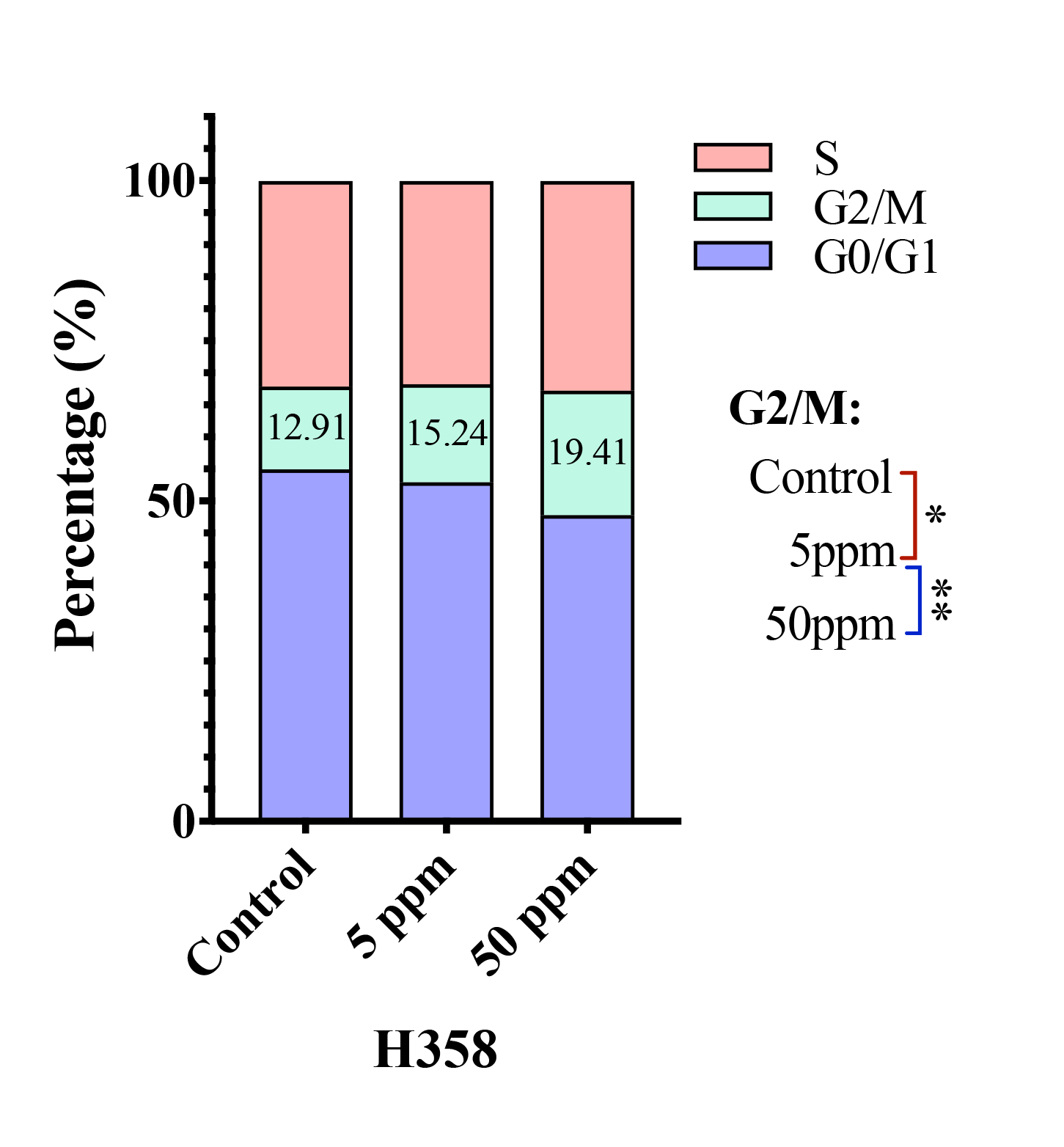
**

**Figure S12.** The proportion of cell division cycle of H358 cells treated with PBS, low-dose (5 ppm), and high-dose (50 ppm) Cu-MOF (n=3, mean ± SD). One-way analysis of variance (ANOVA) was performed: ^**^*p* < 0.01, ^*^*p* < 0.05.


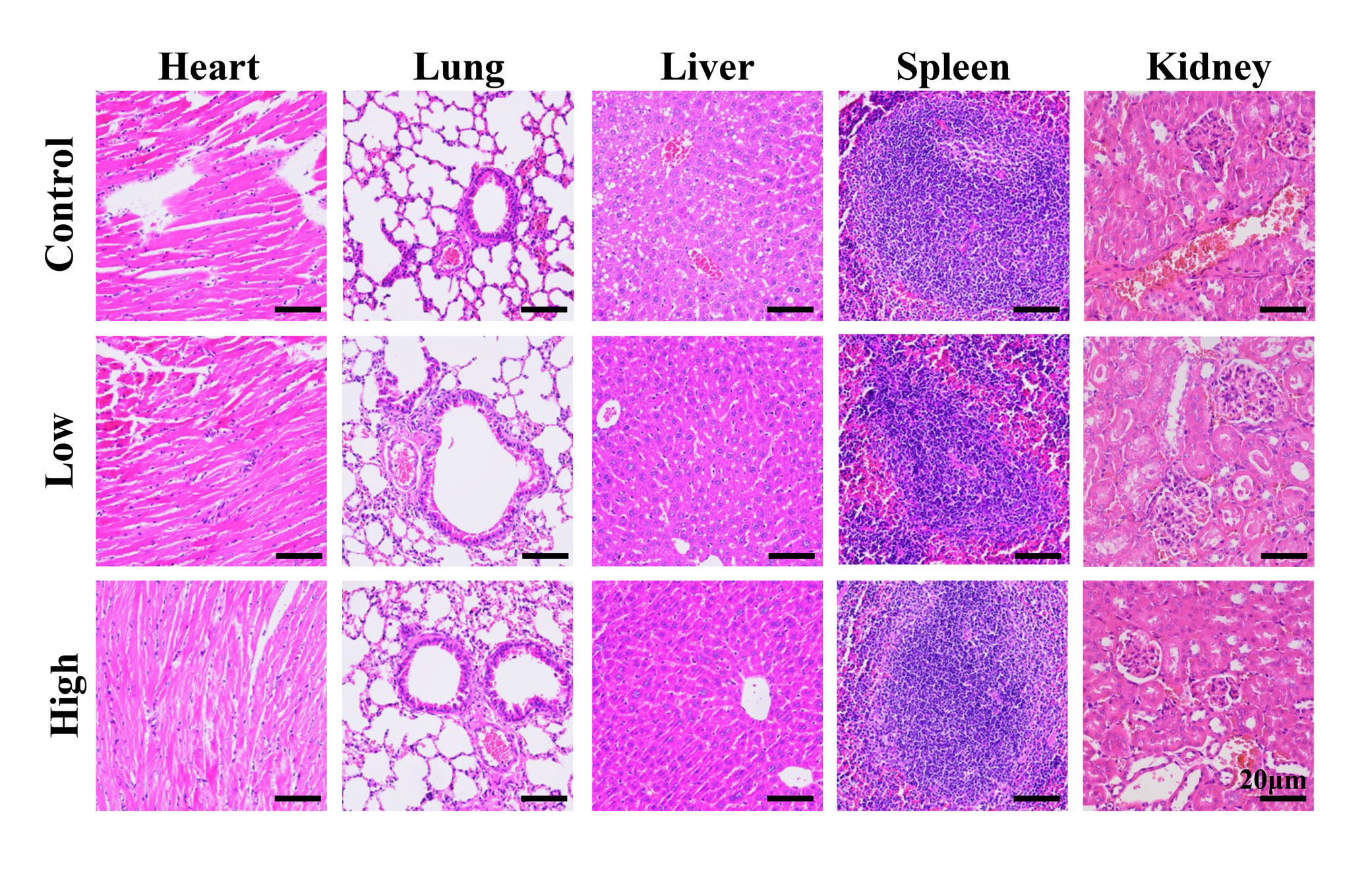


**Figure S13.** H&E staining was performed to evaluate pathological changes in the heart, liver, spleen, lungs, and kidneys of the mice treated with PBS, low-dose (5 ppm), and high-dose (50 ppm) Cu-MOF on the 13th day, showing no significant change of H&E tissue sections. Scale bar = 20 μm.


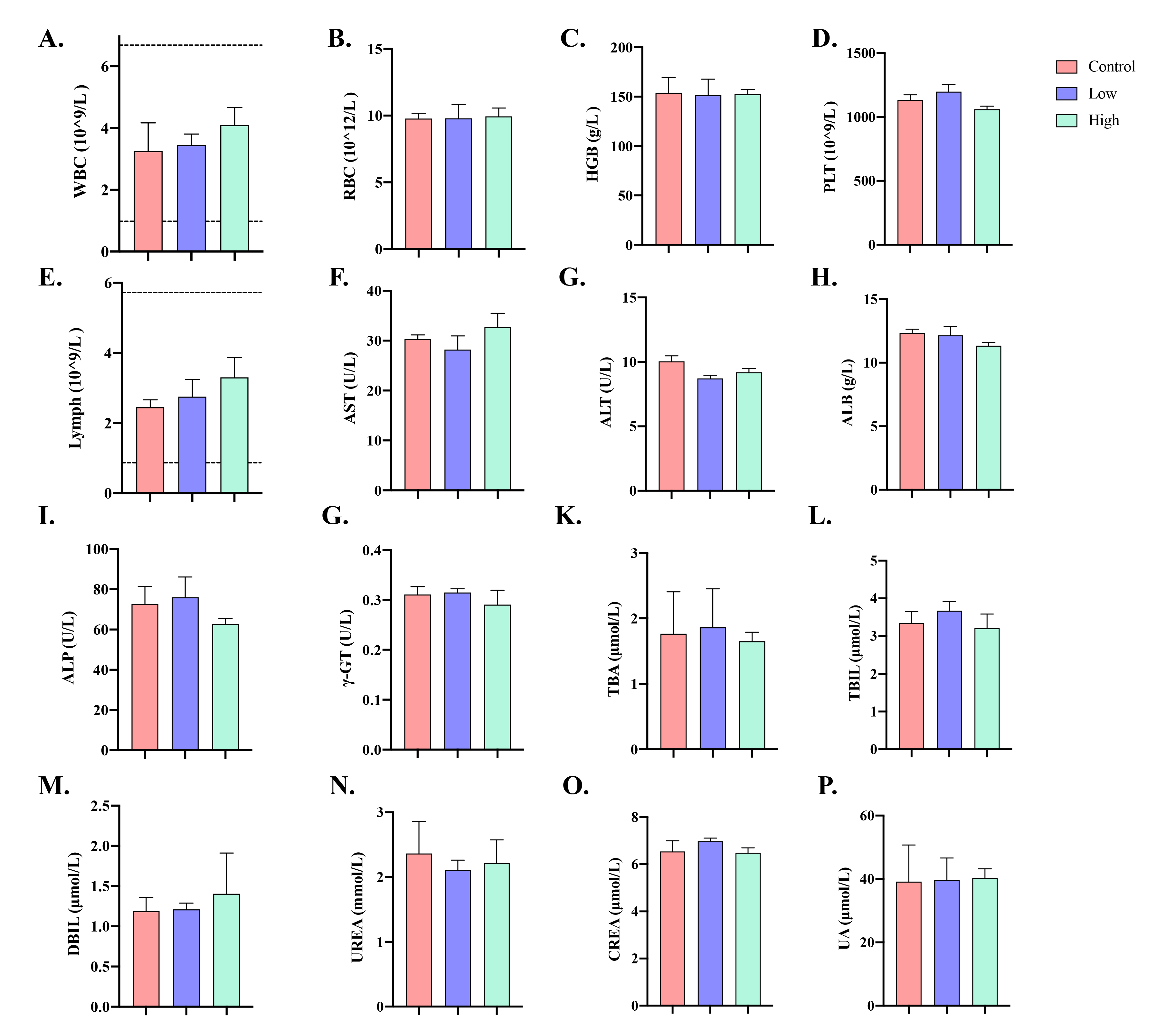


**Figure S14.** Routine blood test and biochemical evaluation of the BALB/c nude mice bearing A549 tumors after different treatments (PBS, low-dose (5ppm), and high-dose (50ppm) Cu-MOF) on the 13^th^ day.


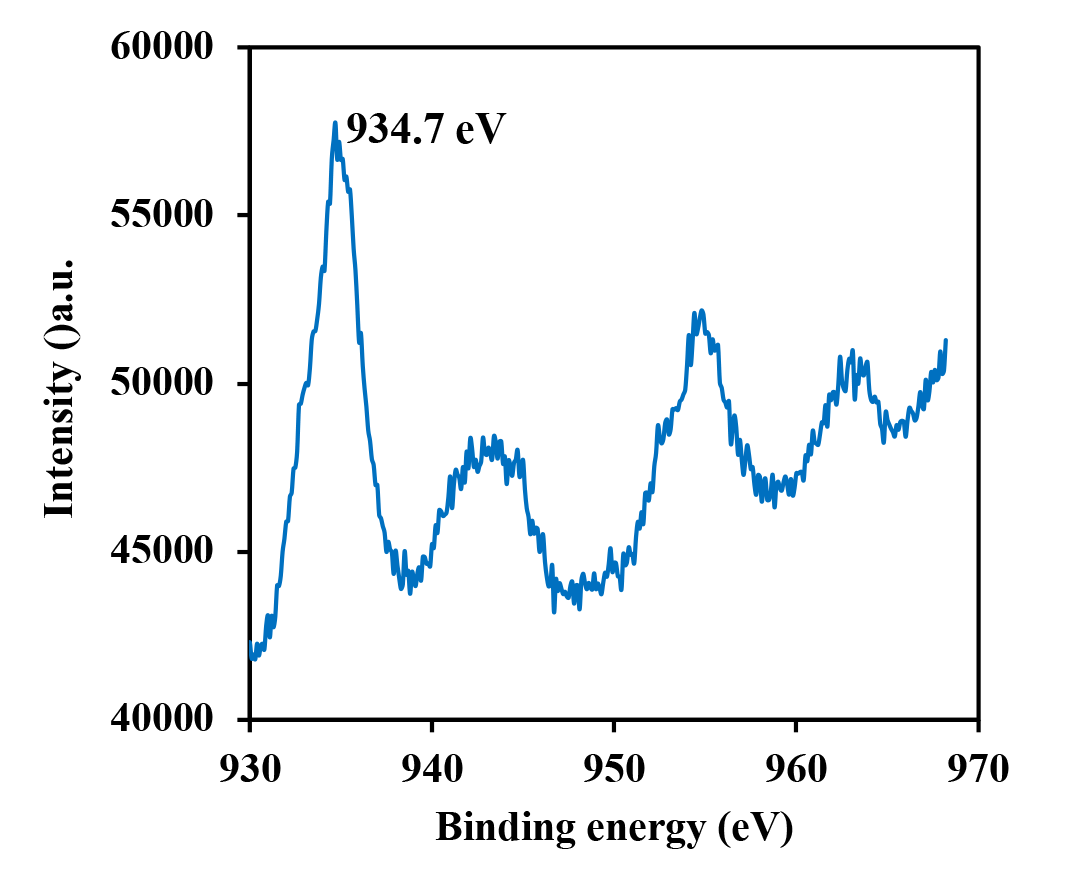


**Figure S15.** The 2p3/2 binding energy of MOF-818. The 2p3/2 binding energy of MOF-818 was 934.7 eV suggesting the valence state of copper in MOF-818 was +2.

**
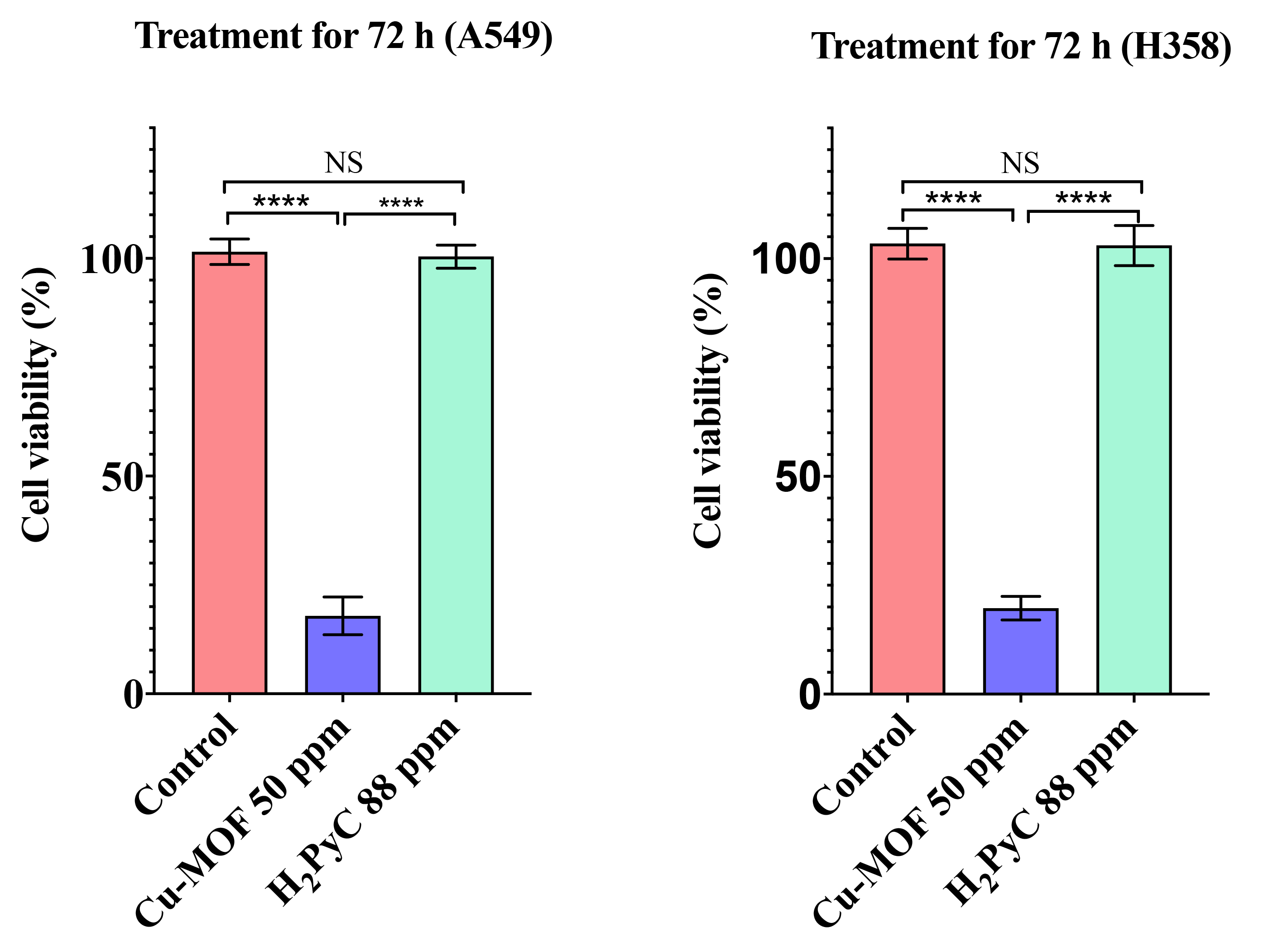
**

**Figure S16.** Cell viability of A549 and H358 cells after various treatments with PBS, Cu-MOF (50 ppm), and H_2_PyC (88 ppm) at 72 h. Student’s t-test was performed: *****p* < 0.0001. NS: No Significance.


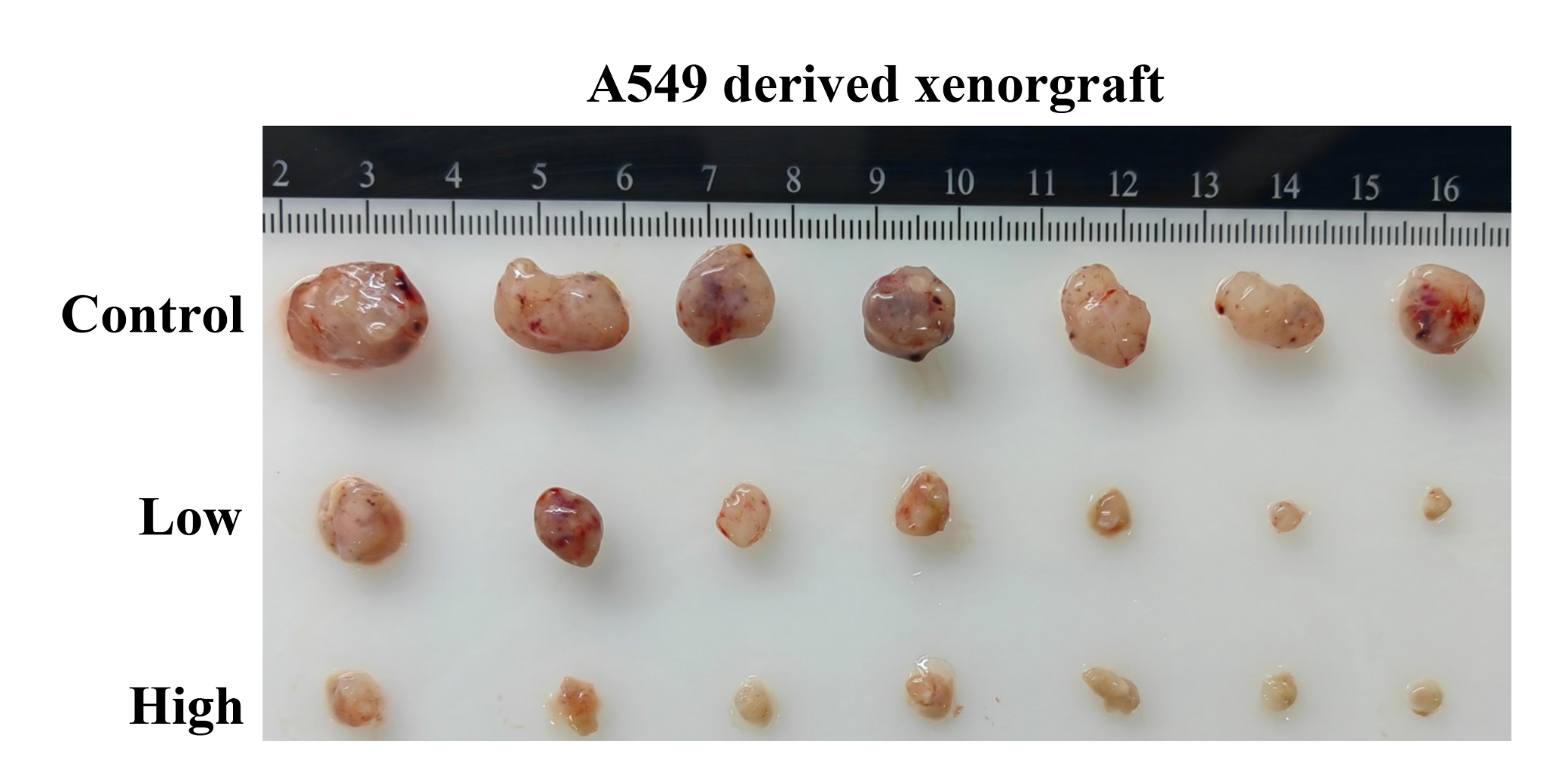


**Figure S17.** Photographs of tumors after treatments with PBS, low-dose (0.05 mg per mouse), and high-dose (0.1 mg per mouse) Cu-MOF (n = 7, mean ± SD).
